# Comparative Transcriptomics of Rice Genotypes with Contrasting Responses to Nitrogen Stress Reveals Genes Influencing Nitrogen Uptake through the Regulation of Root Architecture

**DOI:** 10.3390/ijms21165759

**Published:** 2020-08-11

**Authors:** Prasanta K. Subudhi, Richard S. Garcia, Sapphire Coronejo, Ronald Tapia

**Affiliations:** School of Plant, Environmental, and Soil Sciences, Louisiana State University Agricultural Center, Baton Rouge, LA 70803, USA; rgarcia@agcenter.lsu.edu (R.S.G.); scoronejo@agcenter.lsu.edu (S.C.); ronald.tapia@ufl.edu (R.T.)

**Keywords:** alternate splicing, gene expression, nitrogen use efficiency, *Oryza sativa* L., nitrogen stress, RNA-seq, root architecture, transcription factors, transcriptome sequencing

## Abstract

The indiscriminate use of nitrogenous fertilizers continues unabated for commercial crop production, resulting in air and water pollution. The development of rice varieties with enhanced nitrogen use efficiency (NUE) will require a thorough understanding of the molecular basis of a plant’s response to low nitrogen (N) availability. The global expression profiles of root tissues collected from low and high N treatments at different time points in two rice genotypes, Pokkali and Bengal, with contrasting responses to N stress and contrasting root architectures were examined. Overall, the number of differentially expressed genes (DEGs) in Pokkali (*indica*) was higher than in Bengal (*japonica*) during low N and early N recovery treatments. Most low N DEGs in both genotypes were downregulated whereas early N recovery DEGs were upregulated. Of these, 148 Pokkali-specific DEGs might contribute to Pokkali’s advantage under N stress. These DEGs included transcription factors and transporters and were involved in stress responses, growth and development, regulation, and metabolism. Many DEGs are co-localized with quantitative trait loci (QTL) related to root growth and development, chlorate-resistance, and NUE. Our findings suggest that the superior growth performance of Pokkali under low N conditions could be due to the genetic differences in a diverse set of genes influencing N uptake through the regulation of root architecture.

## 1. Introduction

The foundation of modern agriculture is based on the use of high-yielding cultivars that are responsive to heavy doses of nitrogenous-fertilizers because nitrogen (N) is the single most critical macronutrient used for commercial crop production. The application of nitrogenous fertilizers has doubled with the development of high-yielding cultivars to increase food production for the growing global population [1]. Despite the ecological unsustainability of heavy N fertilization in agriculture, this practice continues unabated. Most applied nitrogen is lost to the environment raising both environmental and health concerns due to air and water pollution [2]. As a result, nitrate contamination of ground and surface water has become both a regulatory and social issue. Over-fertilization with N leads to increased disease incidence and yield loss due to lodging. An increased efficiency of N fertilizers, which is the single largest variable cost for crop production, can enhance the profitability of farming by reducing fertilizer usage [3]. A reduction in use of N fertilizers can be achieved by developing sustainable management practices and crop cultivars with improved nitrogen use efficiency (NUE) to minimize the impact on the global environment [2]. In contrast to the earlier green revolution strategy that focuses on developing genotypes responding to higher doses of N, current research is now directed at genotypes that perform well under N limited environments without compromising crop productivity. In rice growing areas of the USA, an alternate wetting and drying technique is slowly gaining popularity to reduce the cost of cultivation by reducing water usage. However, this strategy will require the development of rice cultivars with improved water and nitrogen use efficiency to reduce N loss during the process. Improvements in N utilization can be enhanced through an improved nitrogen transport, assimilation, and remobilization [4]. Therefore, deciphering the molecular basis of the natural variation for sensing and responding to N availability has great potential to design crop varieties which can ensure a healthy balance between the crop yield and environmental consequences [5,6].

More than half of the world’s population consume rice (*Oryza sativa* L.) as a staple food. Regardless of the physiological and genetic complexity of N stress tolerance, rice is an attractive model organism due to its small genome size and the availability of many genomic and germplasm resources [7,8]. As a result of domestication and artificial selection, there is a significant difference in N uptake and assimilation between the two subspecies of rice [9]. The *indica* varieties have long been known to be superior in NUE compared to *japonica* varieties [10,11]. Based on this premise, Hu et al. (2015) [9] cloned *NTR1.1B*, which explained the divergence in nitrogen use between *indica* and *japonica* subspecies and the superiority of the *indica* allele. Therefore, the mining and utilization of superior variants from *indica* germplasm should help design varieties with improved NUE [12]. Although there were studies focusing on mapping and gene expression in rice, none of these studies involved the USA adapted rice genotypes. Since it is widely known that quantitative trait loci (QTL), gene expression and stability varies with the genetic backgrounds due to the difference in allelic combinations, there is a need to discover the genetic determinants that differentiate US cultivars from the *indica* cultivars for use in rice breeding programs in the United States.

Genetic engineering has been advocated to manipulate nitrogen assimilatory pathway genes [13,14,15,16], but no cultivar has reached farmers’ fields to date despite the claims of an increased biomass and grain yield in transgenic plants. The most plausible approach is to exploit natural genetic variations in existing germplasm [4]. Specifically, the superior ability of *indica* rice for N uptake and mobilization [9,10] offers a unique opportunity to design nitrogen use-efficient cultivars by providing insights into the molecular genetic basis of NUE. However, the real gap between research and commercial product development lies with the difficulty of assembling all beneficial alleles in an advanced breeding line.

RNA-seq is a highly sensitive and powerful transcriptomic tool for identification of differentially expressed genes (DEGs) [17]. It has been applied to identify genes responsive to variable N regime. In most studies, only one genotype is used irrespective of its level of NUE [18,19,20]. Yang et al. [19] identified 1650 DEGs in both root and leaf sheaths under N deficient condition and 86 were transcription factors (TF) from 28 families such as AP2/EREBP, WRKY, GATA, Dof, and MADS. A few studies used comparative RNA-seq approaches using two rice varieties [21,22]. A transcriptome profiling study involving IR64 and N22 under optimal and N starved conditions revealed that most DEGs were associated with starch and chloroplast metabolisms and signaling, and many of these genes were localized within the NUE-related QTL intervals [21]. A comparison of the shoot apical meristem transcriptome between an *indica* variety “YD6” and a *japonica* variety “Nipponbare” under a variable N regime revealed that majority of the DEGs are involved in general stress responses, stimulus responses, and hormonal signaling process [22].

Although many genes involved in N uptake and assimilation are known, information on transcriptional regulation and signal transduction processes, associated with improvements in NUE, is limited. Many QTLs from NUE mapping studies in rice and other crops overlapped with genes that were involved in nitrogen uptake and assimilation [23,24,25]. For a complex trait like NUE, which is controlled by many genes with several interacting environmental factors, we applied a genome-wide expression profiling technique, using a *japonica* genotype “Bengal” and an *indica* genotype “Pokkali”, with contrasting responses to nitrogen availability to discover candidate genes with the potential to improve the NUE in rice. Our analysis revealed many regulatory genes and genes involved in different pathways which can be used for developing strategies to improve the NUE in rice.

## 2. Results

### 2.1. Chlorate Experiment

**Figure 1:**
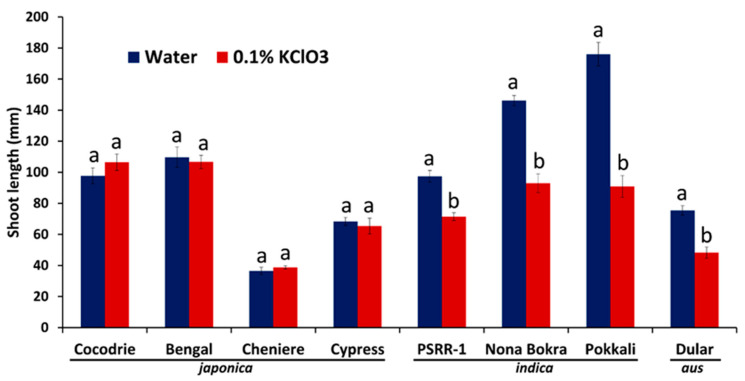
Effect of chlorate uptake on plant height in eight rice genotypes (Cocodrie, Bengal, Cheniere, Cypress, PSRR-1, Nona Bokra, Pokkali, and Dular). The mean seedling heights and standard errors were based on 10 control and 0.1% KCl03-treated seedlings per genotype. Groups are determined by a one-way analysis of variance relative to the respective control at *p* < 0.05. Means with similar letters indicate no significant difference.

Chlorate and nitrate are analogs and the uptake of chlorate during the seedling stage results in growth inhibition due to its toxicity to plants. Hence, the nitrogen uptake capacity was inferred from the shoot length measurements in a chlorate assay. Among the eight genotypes used for this assay, the shoot inhibition rate was significantly higher in *indicaa* genotypes compared with others (Figure 1 and Figure S1). Pokkali and Bengal showed the highest (48%) and the least (2%) shoot reduction, respectively. Based on the result of this initial experiment, Pokkali (*indica*) and Bengal (*japonica*) were selected for N stress and RNA-seq experiments.

### 2.2. Nitrogen Stress Experiment

**Figure 2:**
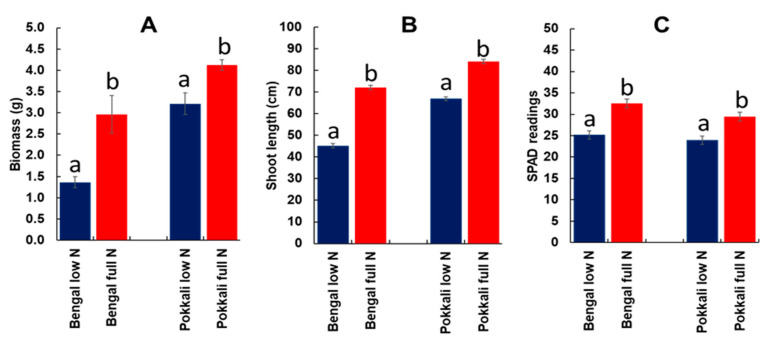
Biomass (**A**), shoot length (**B**), and (**C**) chlorophyll content (SPAD readings) in Pokkali and Bengal under low nitrogen (4 ppm) and full nitrogen (40 ppm) in a hydroponic experiment. Means with similar letters indicate no significant difference at *p* < 0.05 (*n* = 15) determined using a one-way ANOVA.

Limiting the amount of N in nutrient solution was able to differentiate the agro-morphological responses between Pokkali (PK) and Bengal (BG). Generally, a reduction in shoot length, leaf greenness, and biomass was observed in both genotypes (Figure 2). However, Pokkali showed a smaller reduction in shoot length, chlorophyll content, and biomass than Bengal.

**Figure 3:**
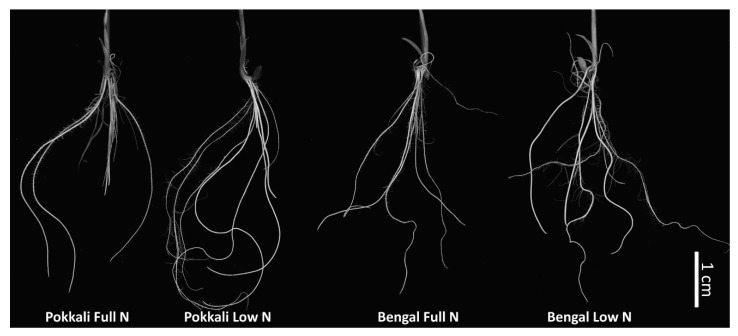
Root architectures of Pokkali and Bengal seedlings under low and full nitrogen treatment.

Changes in root architecture under nitrogen starvation was evident in Pokkali and Bengal (Table 1 and Figure 3). The response to N stress (low N) lead to an increased lateral root length, total number of roots, total root surface area, and root volume except for root diameter/thickness in both genotypes. The root attributes were more pronounced in Pokkali compared with Bengal under normal and low N. The root length and root counts were positively correlated with the total root surface area and root volume (Table S1 and Figure S2).

### 2.3. Transcriptome Analysis under Nitrogen Stress

RNA sequencing of root tissues in different treatments resulted in 421 and 394 million raw reads in Bengal and Pokkali, respectively (Table S2). Treatments were low N (LN), full N (FN) and 1 h after the transfer from low N to full N. All treatments had three biological replicates. The mean assembly percentage to the reference genome was 70 and 83 for Bengal and Pokkali, respectively. Only high-quality reads were used for all downstream analyses. In total, 208,719 and 208,338 transcripts were identified in Bengal and Pokkali, respectively. On average, there were 2652 and 2371 novel transcripts in Bengal and Pokkali, respectively.

**Figure 4:**
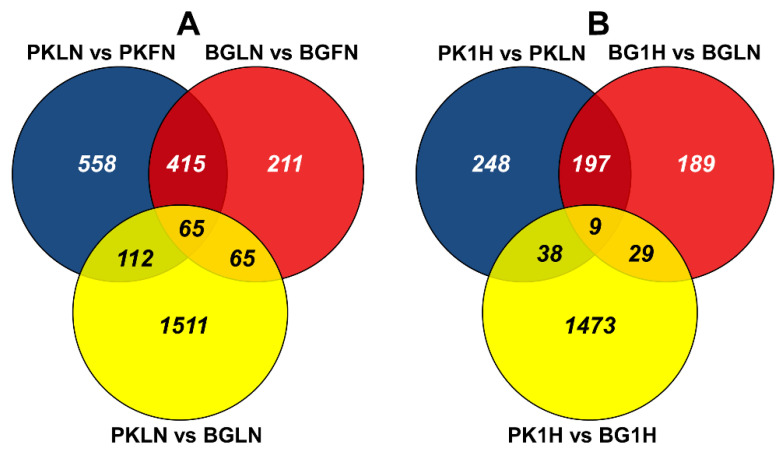
Venn diagrams showing differentially expressed genes (DEGs) in Pokkali and Beng al under low N stress and 1-h recovery; |log2 fold change| ≥ 2 and padj < 0.01). (**A**) Comparison of low N vs. full N, and (**B**) comparison of low N vs. 1 h after transfer to full N. Treatment groups are PKFN (Pokkali-full nitrogen), PKLN (Pokkali-low nitrogen), PK1H (Pokkali 1 h after transfer from low N to full N), BGFN (Bengal-full nitrogen), BGLN (Bengal-low nitrogen), and BG1H (Bengal 1 h after transfer from low N to full N).

**Figure 5:**
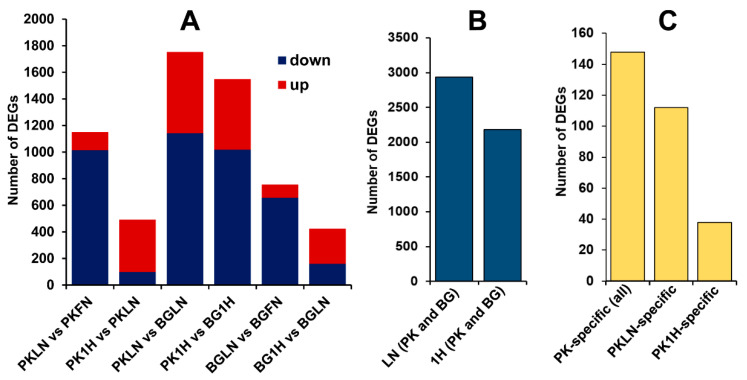
Number of differentially expressed genes in pairwise comparisons of low N and 1-h recovery treatments in Bengal and Pokkali. (**A**) On the stacked bars, blue represents downregulated genes while red represents upregulated genes. (**B**) Combined DEGs of all pairwise comparisons of low N and 1-h recovery treatments in both Bengal (BG) and Pokkali (PK). (**C**) Pokkali-specific DEGs for each treatment. The treatments included in the comparison are PKFN (Pokkali-full nitrogen), PKLN (Pokkali-low nitrogen), PK1H (Pokkali 1-h after transfer from low N to full N), BGFN (Bengal-full nitrogen), BGLN (Bengal-low nitrogen), and BG1H (Bengal 1-h after transfer from low N to full N).

The differentially expressed genes were identified for each pairwise comparisons of Pokkali and Bengal, and N treatments (Figure S3). The number of common, treatment-specific, and genotype-specific DEGs are illustrated in Figure 4 and Figure 5. The total number of DEGs in response to low N was 2937 which was comprised of 1753 (611 upregulated and 1142 downregulated), 1150 (136 upregulated and 1014 downregulated), and 756 (98 upregulated and 658 downregulated) for PKLN (Pokkali-low nitrogen) vs. BGLN (Bengal-low nitrogen), PKLN vs. PKFN (Pokkali-full nitrogen) and BGLN vs. BGFN (Bengal-full nitrogen), respectively (Figure 4A and Figure 5). On the other hand, there were 2183 early N responses (1 h after transfer from low N to full N) DEGs which were comprised of 1549 (531 up and 1018 down), 492 (393 up and 99 down), and 424 (264 up and 160 down) for PK1H (Pokkali 1 h after transfer from low N to full N) vs. BG1H (Bengal 1 h after transfer from low N to full N), PK1H vs. PKLN, and BG1H vs. BGLN, respectively (Figure 4B and Figure 5). Overall, the number of DEGs in Pokkali was higher than in Bengal during low N stress and early recovery. The DEGs in both genotypes grown under low N condition (PKLN vs. PKFN, BGLN vs. BGFN and PKLN vs. BGLN) were mostly downregulated, whereas DEGs were mostly upregulated in early N recovery (PK1H vs. PKLN, BG1H vs. BGLN and PK1H vs. BG1H (Figure 5)).

**Figure 6:**
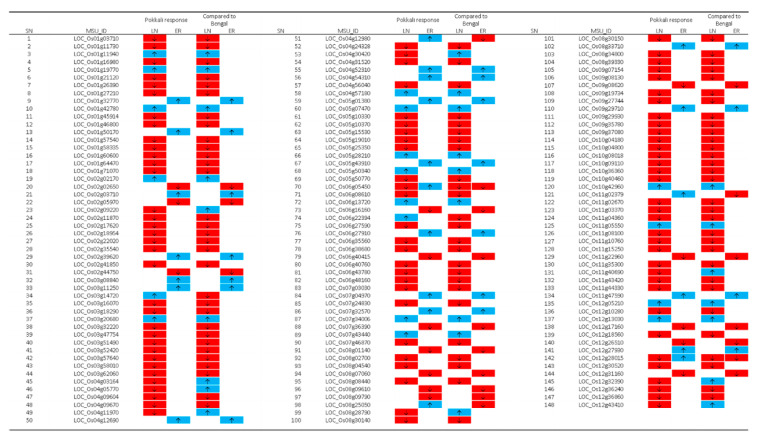
The 148 Pokkali-specific root DEGs under low N and early recovery conditions. The expression pattern of these genes is shown for low nitrogen (LN) and 1-h N recovery/early response (ER) in Pokkali as well as compared to Bengal. The upward arrows in blue blocks and downward arrows in red blocks indicate upregulation and downregulation, respectively.

Furthermore, the Pokkali-specific N response DEGs were 112 (18 up and 94 down), and 38 (23 up and 15 down) for low N [(PKLN vs. PKFN ∩ PKLN vs. BGLN) – (BGLN vs. BGFN)], and early N recovery [(PK1H vs. PKLN ∩ PK1H vs. BG1H) – (BG1H vs. BGLN)], respectively (Figure 5 and Figure 6). Due to the overlapping of some genes, there were 148 Pokkali-specific DEGs (Table S3). Few genes were common between both N conditions. For example, *LOC_Os06g05450* and *LOC_Os12g28015* were both regulated during N stress and early recovery. In addition, a literature review of Pokkali-specific DEGs for gene ontology (GO) functions indicated that 9% of genes were involved in plant growth and development whereas the rest were associated with regulation and metabolism (15%), stress responses (17%) and unknown functions (59% (Figure S4)).

### 2.4. Gene Ontology (GO) and Pathway Analysis of Differentially Expressed Genes under Nitrogen Stress

**Figure 7:**
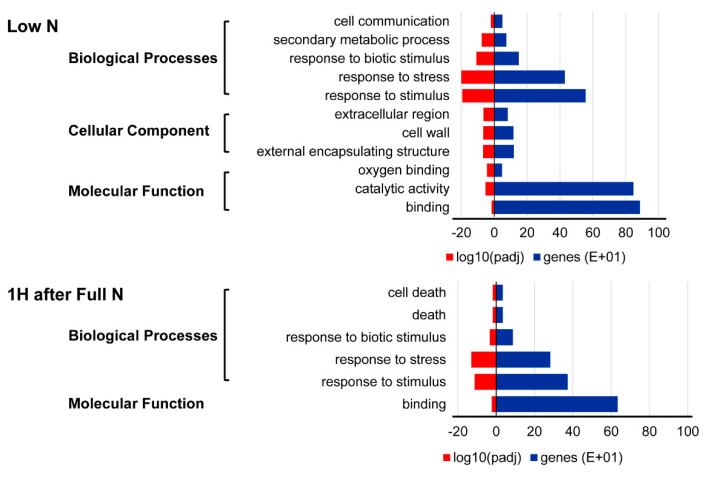
Gene ontology of DEGs from low N and 1-h recovery treatments in Pokkali and Bengal.

GO enrichment was performed to ascertain the functions of DEGs and the related biological processes, whereas the pathway analysis was done to determine the major and specific plant pathways involved in each N treatment. Combined Pokkali and Bengal DEGs from each treatment were used in a singular enrichment analysis (SEA) and Plant Reactome pathway analysis. In the SEA analysis, the N-responsive DEGs from both Pokkali and Bengal were classified within the three domains: “biological process”, “molecular function” and “cellular component” (Figure 7). There were 11 and 6 significant GO terms identified for low N and early N recovery, respectively. The “biological process” had the greatest number of enriched GO terms among the three classified domains, and this was true for both low and recovery N treatments. The “molecular function” domain revealed “oxygen binding”, “binding”, and “catalytic activity” GO terms in low N while the early N response showed “binding” GO terms. These differences in GO terms showed diversity in response to different N conditions. In addition, the GO enrichment also revealed that only low N conditions had the “cellular component” terms: “the extracellular region”, “external encapsulating structure”, and “cell wall”. These confer a root morphological response for foraging N under low N condition as indicated by root induction.

Plant Reactome pathway analyses of DEGs showed major plant pathways involved in “cellular process”, “growth and developmental process”, “metabolism and regulation”, “abiotic and biotic stimuli and stresses” (Table 2). The majority of the DEGs in low N and early N recovery (84% and 81%, respectively) were involved in the “metabolism and regulation” plant pathway (Figure S5). The common basic “metabolism and regulation” pathways between low N and immediate recovery conditions were hormone signaling, transport and metabolism, amino acid metabolism, carbohydrate metabolism, cofactor biosynthesis, cytokinin biosynthesis, inorganic nutrient metabolism, and secondary metabolism. Similarly, the “cellular process”, “growth and developmental process” and “response to abiotic and stimuli and stresses” had common basic pathways as well. In addition, unique pathways for each N response were observed: vegetative structure developments, amine and polyamine biosynthesis, detoxification, fatty acid and lipid metabolism, generation of precursor metabolites and energy, photorespiration, response to phosphate deficiency, recognition of fungal and bacterial pathogens and immunity response for a low N response, and DNA replication and activation of the pre-replicative complex for early N recovery. This indicates that there are common basic pathways for both Pokkali and Bengal in response to low N and early N recovery and also unique pathways to cope with each N condition.

### 2.5. Expression Pattern of Nitrogen Utilization and Long Distance Signaling

The nitrogen availability in the nutrient solution affected certain N utilization genes in the roots of Pokkali and Bengal which were essential for N uptake, translocation, and assimilation. Some of these genes were found to be differentially expressed (Table 3). The N uptake genes were nitrate reductases (*LOC_Os08g36480*, *LOC_Os08g36500*, and *LOC_Os02g53130*). *Nitrate reductase 1* and *2* were not differentially expressed in Pokkali, regardless of N conditions. However, these reductases in Bengal were upregulated at early recovery. The *NADH/NADPH-dependent NO_3_-reductase 2* was differentially expressed in Pokkali and Bengal at low N, and recovery and both cultivars showed similar regulation pattern. Among the N transport/translocation-related genes, the ammonium transporter (*LOC_Os02g40730*) and high-affinity nitrate transporter 2.1 (*LOC_Os02g02170*) showed different expression patterns in Pokkali and Bengal under low N conditions, whereas the other nitrate transporter genes (*LOC_Os10g40600*, *LOC_Os02g02190,* and *LOC_Os01g50820*) showed similar patterns for both cultivars. Lastly, a few N assimilation genes, such as glutamine synthetase 1;2 (*LOC_Os03g12290*) and NADH-GOGAT (*LOC_Os01g48960*), were regulated in Pokkali and Bengal with similar expression patterns.

Certain proteins such as C-terminally encoded peptides (CEPs) and CLV3/ESR-related (CLE) peptides were known to be involved in long distance systemic signaling in plants during N deprivation [26]. Long distance signaling between the shoot and roots is important in the root growth response during N stresses. In our study, a few CEPs (*LOC_Os05g11620*, *LOC_Os05g11580,* and *LOC_Os06g43080*) and CLEs (*LOC_Os01g23705*, *LOC_Os01g32560* and *LOC_Os03g48570*) showed differential expressions in Pokkali and Bengal roots at various N treatments (Table S4).

### 2.6. Transcription Factors and Signaling for Nitrogen Utilization and Root Growth and Development Genes

Transcription factors are known to control plant growth and modulate responses to biotic and abiotic stimuli through the regulation of gene expression. There were 152 and 125 TFs encompassing 30 TF families identified in root tissues in Pokkali and Bengal, respectively (Table 4). The general view of the regulation pattern of TFs indicated that most of the TFs were downregulated in low N conditions while they are upregulated in early N recovery stage (Figure S6). An analysis of DEGs from different transcription factor families revealed that members of ethylene responsive factor (ERF), WRKY, basic helix-loop-helix (bHLH), MYB, C2H2 zinc finger, and NAC families were most abundant with 14.9%, 14.4%, 8.9%, 8.9%, 7.9% and 7.9% of TFs, respectively (Figure S7).

### 2.7. Phytohormones Related to N Stress and Root Growth and Development

**Figure 8:**
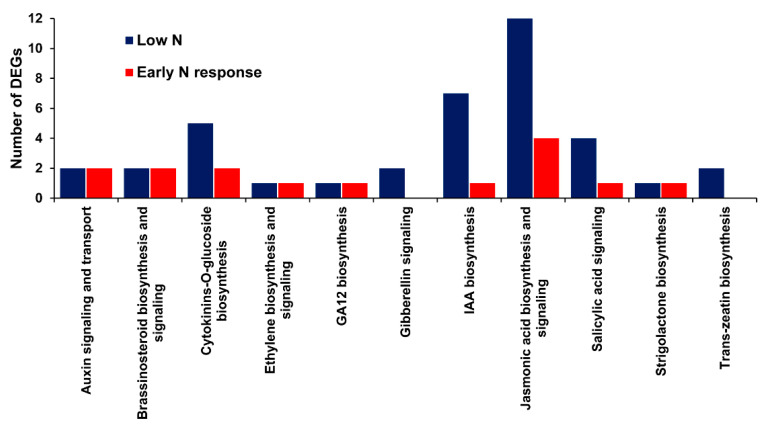
Number of DEGs identified related to phytohormone signaling during various nitrogen conditions. The plant hormone signaling genes identified were obtained from the combined data of low N (PKLN vs. PKFN, PKLN vs. BGLN and BGLN vs. BGFN) and 1-h response/early N response (PK1H vs. PKLN, PK1H vs. BG1H and BG1H vs. BGLN).

Plant hormones play a significant role in the regulation of plant development and environmental responses. Based on Plant Reactome annotations, several DEGs with phytohormone signaling functions in various N conditions were identified, suggesting the important role of plant hormones in processes involved in improving the NUE (Figure 8).

### 2.8. Differentially Expressed Genes Overlapping the Nitrogen Stress-Related QTL

**Figure 9:**
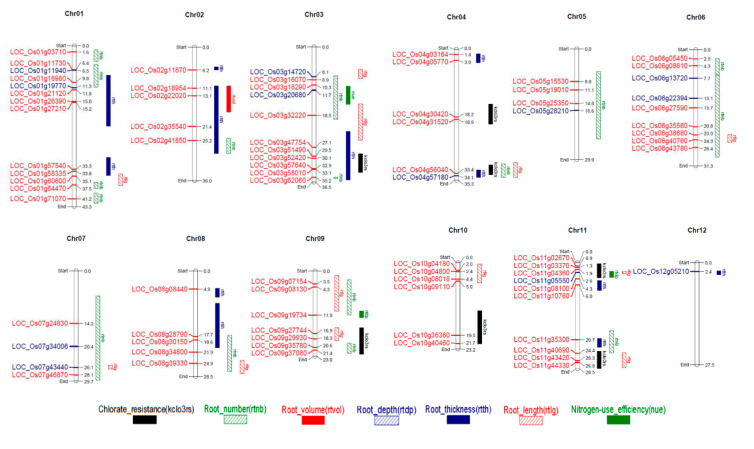
Pokkali-specific low N response DEGs co-localized on chromosomes with chlorate resistance, root development and nitrogen use efficiency (NUE) quantitative trait loci (QTL). Locus names in red font: downregulated, blue font: upregulated. QTLs included were chlorate resistance (kclo3rs): black solid bar; root number (rtnb): green diagonal bar; root volume (rtvol): red solid bar; root depth (rtdp): blue diagonal bar; root thickness (rtth): blue solid bar and root length (rtlg): red diagonal bar.

We found several DEGs co-localized with the selected QTLs for low N responses (Table S5), and only 81 of these were Pokkali-specific (Figure 9). These co-localized DEGs consisted of loci that lie within, and some overlapping with other QTLs. For example, *LOC_Os03g20680* lies within NUE and root number (rtnb) QTLs and *LOC_Os11g40690* lie within chlorate resistance (kclo3rs), rtnb, and root length (rtlg) QTLs.

### 2.9. Alternate Splicing Events under Nitrogen Stress

**Figure 10:**
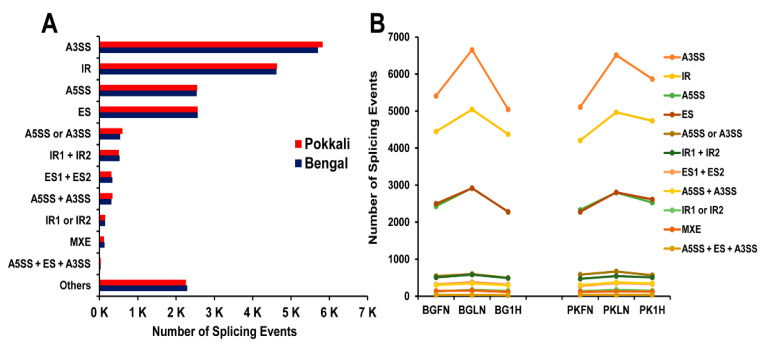
Annotation of alternate splicing (AS) events in the root transcriptome of rice. (**A**) Distribution of the various AS events in combined transcript assembly of all treatments in Pokkali (PK) and Bengal (BG). Others refer to complex events. (**B**) AS events are in the combined transcript assembly of each genotype and treatment combination.

A total of 39,668 alternative splicing (AS) events were identified in Bengal (19,753) and Pokkali (19,916) (Figure 10A). The most predominant AS event was A3SS with nearly 29% of total events in both genotypes followed by intron retention (IR (BG: 23.4%, PK: 23.3%)), exon skipping (ES (BG: 13.0%, PK: 12.9%)), alternative 5′ splice site (A5SS (BG:12.9%, PK:12.8%)), A5SS or alternative 3′ splice site (A3SS (BG: 2.8%, PK:3.0%)), IR1 + IR2 (BG: 2.7%, PK: 2.5%), ES1 + ES2 (BG: 1.7%, PK: 1.6%), A5SS + A3SS (BG: 1.6%, PK: 1.7%), mutually exclusive exon (MXE (BG: 0.7%, PK: 0.6%)), IR1 or IR2 (BG: 0.7%, PK: 0.8%), and A5SS + ES + A3SS (0.2%).The analysis of the combined assembly of genotypes and treatments showed that Bengal had slightly more AS events in FN and LN treatments compared to Pokkali while Pokkali had higher AS in 1 h after recovery than Bengal (Figure 10B). Looking at the genotype per treatment comparison, Bengal shows the highest number of AS events in low nitrogen treatment followed by a decrease in 1-h recovery treatment. Out of the 148 Pokkali-specific differentially expressed genes, 17 exhibited alternative splicing events (Table S6). Overall, there were differences in the types of AS events between different treatments within a genotype or between both genotypes.

### 2.10. Gene Expression Validation of Selected Genes by Quantitative Reverse Transcription PCR (qRT-PCR)

Eleven genes were selected based on their response to nitrogen stress, growth, yield, and root development to validate the expression pattern obtained from RNA-seq data using qRT-PCR under different treatments (full N, low N, and 1-h recovery) in both genotypes. After normalizing the Ct values using the internal control gene *EF1α* (Figure S8), a fold change calculation was used for a comparison between genotypes (i.e., full nitrogen treatment as a reference sample for low nitrogen while low nitrogen treatment was used as reference sample for 1-h recovery treatment). Pearson’s correlation coefficient (*r =* 0.71), based on the log_2_ fold change expression values of the selected genes, showed a positive and linear relationship between qRT-PCR and RNA-seq data indicating the reliability of RNA-seq results (Figure S9).

## 3. Discussion

Since a bulk of N from the applied fertilizers is lost to environment, reducing fertilizer use is necessary for sustainable agriculture. This can be accomplished by enhancing crop plants’ ability to efficiently uptake and assimilate N so that yield can be maintained with reduced fertilizer application. Therefore, an improved understanding of plants’ response N stress condition at the molecular level is needed to make such genetic improvements. In rice, RNA-seq studies have been conducted to reveal differentially regulated genes related to tillering [22] and carbon and nitrogen metabolism [20] in response to nitrogen availability. In this study, we used an *indica* genotype “Pokkali” and a *japonica* genotype “Bengal” to focus on root characteristics due to its pivotal role in N uptake [27]. We applied a comparative transcriptomic approach to differentiate transcriptional responses in the root tissues of these two genotypes under chronic nitrogen stress, as well as after the transfer to the optimal nitrogen condition and to identify differentially expressed genes which may have the potential to improve NUE. We hypothesize that variations in the nitrogen uptake capacity are due to the differential expression of genes which regulate the root architecture in plants.

Our results on chlorate assay (Figure 1) and seedling parameters (Figure 2 and Figure 3) showed a clear superiority of *indica* genotypes over *japonica* genotypes, with respect to nitrogen uptake and utilization thus confirming earlier studies [9,12,28]. These results prompted us to use Pokkali (*indica*) and Bengal (*japonica*) for a transcriptomic analysis of root tissues. The chlorate assay was supplemented with the N starvation study that showed a better agro-morphological response in Pokkali compared to Bengal. Pokkali was earlier reported to have a higher NUE [29]. Responses to nitrogen stress brought about changes in the root morphology and expression of genes related to nitrogen uptake and/or assimilation. A previous study on root architectural changes in response to variable N applications in different rice genotypes demonstrated the importance of root length and root number in enhancing the N foraging ability [30]. Hence, the low nitrogen availability induced root growth and development promoting more and longer lateral roots which resulted in a higher total root surface area and volume in Pokkali compared to Bengal. Our observations on the involvement of genes in root growth and development, as well as their overlapping with the QTL regions controlling root architecture, indicated that these genes may be responsible for the efficient uptake of N under the limited nitrogen condition in Pokkali.

Among the DEGs, there were several TFs and signaling genes involved in the root growth and development under low N conditions. Transcription factors belonging to the ERFs, WRKYs, bHLHs, MYBs, CH2H2s, and NACs play a multifaceted and overlapping role in the regulation of cell growth, development and proliferation, phytohormone signaling, biotic and abiotic stimuli/stress responses, regulation, and metabolism [31,32,33,34,35,36]. The genes involved in phytohormone signaling proteins/pathways control physiological and morphological responses under N stress [37,38]. Cytokinins, auxins, and gibberellin were involved in the network of nitrate-signaling pathways in plant developmental processes [39]. In addition, there were proteins such as CEPs and CLEs which were regulated under N stress and recovery. These proteins were involved in long distance shoot-to-root signaling and the systemic responses of root growth and development, specifically during N stress [26,39,40]. Some CEPs regulate the expression of *NRT2.1* in *Arabidopsis*, suggesting interactions with core N uptake apparatus [41]. Therefore, there may be crosstalk between the physiological (nitrate transport) and morphological (root) responses under N stress and recovery involving phytohormone signaling genes, TFs, signal proteins, and transporters [26,39].

Examination of the root DEGs between Pokkali and Bengal showed more genes in Pokkali than in Bengal under N starvation. Likewise, the number of DEGs during early N recovery was also higher in Pokkali than in Bengal roots. It was not just the involvement of more DEGs, but also that the allelic differences resulting from different genetic backgrounds may be responsible for an increased N uptake efficiency in Pokkali compared to Bengal. There is a sizable number of genes overlapping between both genotypes observed in our study compared to an earlier study [21]. These common genes may be involved in the inherent mechanisms associated with uptake regardless of genotypic differences.

Nitrogen stress conditions caused *NRT2.1* (*LOC_Os02g02170*) to be profoundly more modulated in Pokkali than in Bengal roots, while the nitrogen assimilation-mobilization genes, such as ammonia transporters, nitrate reductases, glutamine synthetases, and glutamine oxoglutarate aminotransferases (GOGAT), were either unchanged or downregulated under low N conditions (Table 3). The nitrate transporters in Pokkali played a major role in enhancing the uptake of N, particularly under limited N conditions, compared to Bengal. In addition, the N assimilation-mobilization activity was reduced as the latter processes were highly regulated by feedback inhibition depending on the nitrogen availability of plants. The downregulation of nitrate reductase, ammonia transporters, and glutamine synthetase could be due to the regulation of the substrate for nitrogen assimilation, particularly during chronic N stress.

There were several Pokkali-specific DEGs that might have contributed to Pokkali’s advantage over Bengal under both low and optimal N conditions. These DEGs were involved in stress responses, growth and development, regulation and metabolism. Some of these DEGs co-localized with QTLs related to root growth and development, chlorate-resistance, and NUE. These observations suggested that a combination of physiological and root morphological adaptations are responsible for N uptake by roots in rice. This has been demonstrated in case of the *OsNRT2.1* which was involved in NO_3_^−^ uptake under a low external NO_3_^−^ concentration [42] and during root development [26,43]. The *OsNRT2.1*-overexpressing rice plant not only enhanced vegetative growth under low N conditions [42], but also enhanced the yield compared to the wild type under alternating wet and dry conditions [44] and drought stress [45] by increasing nitrogen uptake. This gene also promoted root elongation via auxin transport [43]. Although both genotypes promoted root architecture systems that could have aided in N foraging under low N, the root architecture is more pronounced in Pokkali than in Bengal. Another gene, *OsDUR3* (*LOC_Os10g42960*), a high-affinity urea transporter, was upregulated under low N, confirming earlier studies [21,46]. Since *OsDUR3* improved the rice yield under nitrogen deficient conditions by increasing urea uptake [47], the increased expressions of *OsDUR3* should help in N foraging. Therefore, the upregulation of both *OsDUR3* and *OsNRT2.1* may be enhancing N foraging in the form of either nitrate or urea.

Two important Pokkali-specific differentially expressed genes were *Nitrate-Inducible, GARP-type Transcriptional Repressor 1 (NIGT1, LOC_Os02g22020*) and *GA insensitive dwarf1* (*GID1, LOC_Os03g57640*), which were involved in nitrogen responses in rice through GA signaling [48,49]. *NIGT1* overexpression affected some genes including *GID1* [48], which overlapped with our DEGs under low N. Since *NIGT1* is a negative regulator of *NRT2.1* [50], the downregulation of *NIGT1* under N stress and upregulation after the transfer to optimal nitrogen conditions was expected due to an increased availability of nitrate. This observation suggests the superiority of the Pokkali allele in enhancing N uptake under N stress. A recent study discovered the role of *GID1* and *nitrogen-mediated tiller growth response 5* (*NGR5* (*LOC_Os5g32270*)) in gibberellin signaling by nitrogen fertilization to enhance tillering in rice [49]. *NGR5,* an APETALA2-domain transcription factor responsible for recruiting polycomb repressive complex 2 (PRC2) to regulate the transcription of tillering repressing genes, is dependent on nitrogen application. Semi-dwarf rice strains resist lodging under heavy N fertilization resulting in high yield despite the accumulation of growth repressing Della proteins whose degradation was regulated by *GID1*. Similarly, *NGR5* is also targeted by *GID1* for degradation. Although *NGR5* was not among the Pokkali-specific DEGs, it was differentially expressed in other pairwise comparisons. Since there is a competition for the involvement of *GID1* in both Della and NGR5 degradation, further investigation is needed to clarify these competitive interactions and how to modulate these interactions to enhance the grain yield of rice with reduced N fertilizer applications.

In our study, the alternative 3′ splice site (A3SS) was the major AS category which contrasts with intron retention reported in other studies in rice [51,52]. The number of AS events was more in the N deficient condition compared to the optimal N condition whereas no significant difference between two conditions was reported in an earlier study [19]. The 17 Pokkali-specific DEGs having AS events included transcription factors, stress-related genes, chloroplast precursor genes, and several key enzymes such as *BIM2,* zinc finger C3H4 type domain containing protein, pentatricopeptide (PPR) repeat containing protein, eukaryotic aspartyl protease domain containing protein, indole-3-glycerol phosphate synthase, isochorismate synthase 1, hydrolases, transferases, dehydrogenases (Table S6). All these results indicate the possible role of AS in regulating gene expressions for stress adaptation [53,54].

BIM2 (*LOC_Os09g29930*) is a bHLH type transcription factor that controls cell proliferation to cell lineage establishment [33]. Alternate splicing in this gene was only observed in Pokkali. It was downregulated and co-localized with root length QTL (rtlg; QTL ID: AQHE006) [55] and chlorate resistance QTL (kclo3rs; QTL ID: AQF102) [55], indicating its possible role in N uptake through the modulation of root architecture. Its involvement in brassinosteroid-regulated gene expression, that affects growth in *Arabidopsis,* further supports its role [56]. The late embryogenesis abundant protein, *OsLEA3–2* (*LOC_Os03g20680*) is another Pokkali-specific DEG showing AS, which imparts tolerance to salt and osmotic stress [57]. It is highly upregulated in this study under N stress in Pokkali when compared to Bengal. Its co-localization to the QTL regions of NUE (*qNUE-3*) [58] and root number (rtnb; QTL ID: AQEL011) [55] suggests the importance of this gene during low N stress conditions.

Cinnamyl alcohol dehydrogenase 4 (*LOC_Os11g40690*), a key enzyme in the biosynthesis of an important cell wall component lignin [59], was downregulated in low N treatment in Pokkali root tissues, but its expression was higher than it was in Bengal. Pokkali’s superiority in developing an efficient root system architecture under low N conditions was further strengthened due to the co-localization of this gene with the QTLs for root number (QTL ID: CQAW29), root length (QTL ID: AQAR002), and chlorate resistance (QTL ID: AQF105) [55].

The *LOC_Os02g09220* (cytochrome P450) showed the same expression pattern as cinnamyl alcohol dehydrogenase 4. It may be involved in maintaining plant growth under N stress due to its essential role in regulating growth and enhancing stress tolerance through the mediation of gibberellin homeostasis [60]. GDSL-like lipases, that are involved in biotic [61] and abiotic stress tolerance [62], were differentially expressed in our study as well as in a previous study [21]. Besides, Pokkali-specific DEGs included many disease resistance genes (*LOC_Os08g28790; LOC_Os05g25350, LOC_Os10g04180, LOC_Os12g36860*), which supports the notion of crosstalk as a common phenomenon for plants’ adaptation under different biotic and abiotic stress conditions including nitrogen stress.

## 4. Materials and Methods

### 4.1. Plant Materials and Cultivation

Plant materials for the preliminary screening of NUE included four *japonica* (Cocodrie, Bengal, Cheniere, and Cypress), three *indica* rice genotypes (PSRR-1, Nona Bokra, and Pokkali), and one *aus* genotype (Dular). All *japonica* genotypes were high-yielding cultivars released by LSU Agricultural Center. Dular is a lowland drought tolerant cultivar with deep roots [63]; Nona Bokra and Pokkali are salt tolerant land races [64,65]. PSRR-1 is a weedy rice genotype from Louisiana [66] and is closely related to *indica* subspecies [67].

### 4.2. Chlorate Assay

A chlorate assay was initially performed to screen for the nitrogen uptake capacity of eight genotypes in hydroponic experiments following Teng et al. [68] with two treatments: (a) control (no chlorate), and (b) chlorate-treatment (0.1% potassium chlorate solution). For plant materials, 100 seeds from each test cultivar were sanitized by a soaking in 5% hypochlorite for 30 min followed by rinsing with sterilized water. Seeds were germinated in petri-plates for 5 days at 28 °C. Ten seedlings with uniform growth per genotype were transferred into the hydroponics set-up; three replications and shoot lengths were measured after 7 days. A one-way ANOVA (alpha = 0.05) was performed to assess the differences in responses among cultivars and treatments.

### 4.3. Nitrogen Stress Response Experiment

A nitrogen stress experiment was performed to measure the agronomic response of Pokkali (*indica*) and Bengal (*japonica*). It involved plants grown in a hydroponic set-up with a Yoshida nutrient solution in a semi-controlled environment. Two treatments were set up: (1) low nitrogen (LN), which contains one tenth of the normal amount of nitrogen (4 ppm of N) in Yoshida solution, and (2) full nitrogen (FN), which contains the normal amount of nitrogen (40 ppm of N). Seed sanitization and germination were similar to the chlorate assay mentioned earlier. There were three replicates for each treatment. Plant measurements were performed at 30 days after transferring to hydroponics. Five plants for each replicate were sampled for the measurement of shoot lengths, root-shoot fresh and dry weight, and chlorophyll content/leaf greenness. After taking the fresh weight, shoots and roots were oven-dried at 60 °C for one week to measure the dry weight. Chlorophyll measurements were performed using a SPAD meter (SPAD 502 Chlorophyll Meter, Spectrum Technologies, Inc., Aurora, IL, USA). The percent reduction in the shoot length, biomass, and chlorophyll content in low N compared to full N was calculated. All phenotypic data were subjected to a Student’s T-test (alpha = 0.05) to assess the difference in response to LN and FN between Pokkali and Bengal.

Intact root samples from at LN and FN treatment were collected and scanned using a Xerox^®^Altalink^®^ C8035 (600 × 600 dpi) scanner. Image analyses were performed using the plug-in SmartRoot [69] in Image J [70]. Root architecture parameters such as the root length, root diameter/thickness, root number, root volume and surface area, were measured. A one-way ANOVA was conducted to identify significant differences between LN and FN treatments, and between Pokkali and Bengal.

### 4.4. Hydroponic Experiment for RNA-Seq

Both parents were grown in a hydroponic setup under FN and LN, like the nitrogen stress experiment, and the seed preparation and cultivation conditions were also identical. Twenty seedlings were allocated for FN and 40 seedlings for LN. Root samples were collected at the 4-leaf stage around 14 days after transferring to hydroponics. Ten plants were collected and pooled for each replicate of the FN and LN treatment per cultivar. The remaining plants from the LN treatment were transferred into the FN treatment and then ten samples were collected and pooled for each replicate at a 1 h time point after transfer (recovery experiment). Collected root tissues were placed in liquid N during collection and stored in a −80 °C freezer until RNA extraction.

### 4.5. RNA Isolation, Library Preparation, and Sequencing

The total RNA from root tissues of three biological replicates per treatment was isolated using RNApro™ Solution (MP Biomedicals, Santa Ana, CA, USA). The quality and quantity of RNA were evaluated using an ND-1000 Spectrophotometer (Thermofisher Scientific, Waltham, MA, USA) and were then treated with PerfeCTa DNase I (Quantabio, Beverly, MA, USA). Purified RNA samples were shipped to Novogene Co. Ltd. (Sacramento, CA, USA) for quality checking using an Agilent 2100 Bioanalyzer (Agilent Technologies, Santa Clara, CA, USA), library preparation, and sequencing using an Illumina HiSeq platform with a paired-end 150 bp sequencing strategy.

### 4.6. Reference Genome-Based Mapping

The quality of the raw reads was checked using FastQC (version 0.11.7, Babraham Bioinformatics, Cambridge, UK) with Phred quality score reads of ≥ 30 used in downstream analyses. Subsequent bioinformatics analyses were performed using high performance computing resources provided by Louisiana State University (http://www.hpc.lsu.edu). The processed paired-end reads were mapped to the Nipponbare reference genome (Os-Nipponbare-Reference-IRGSP 1.0) [71]) using HISAT2 (v2.0.1) [72]. All raw read sequences of Bengal and Pokkali were deposited in the NCBI’s sequence read archive (SRA) under the accession number PRJNA644654.

### 4.7. Transcript Assembly and Analysis of Alternative Splicing Events

The reference-based assembly of transcripts for each sample mapped with HISAT2 was done with Stringtie v.1.3.3 using the default settings [73]. The merging of transcript assemblies for each genotype per treatment was done with Stringtie using the merge option. The resulting GTF files were then processed in AStalavista v4.0 [74] for alternative splicing (AS) event identification. The analysis of these AS events was done following the method of Sammeth et al. [75], which identifies events such as intron retention (IR; 0,1^2-), exon skipping (ES; 0,1-2^), alternative 3′ splice sites (A3SS; 1-,2-), alternative 5′ splice sites (A5SS; 1^,2^), mutually exclusive exon (MXE; 1-2^,3-4^), and complex events (i.e., A5SS + A3SS, A5SS + ES + A3SS, etc.).

### 4.8. Differential Expression, Genotype and Treatment Comparison, and Ontology

Mapped reads were processed with featureCounts [76] to get the raw count table for genes in each sample. Genes with less than 10 reads across all samples were removed from downstream analyses. Log2 fold changes (LFC) were estimated using the DESeq2 R package [77] wherein genes with LFCs ≥ 2 (upregulated), ≤ 2 (downregulated), and a Benjamin–Hochberg adjusted *p*-value of <0.01 were considered as differentially expressed genes.

The expression profiles of Pokkali and Bengal in response to variable nitrogen treatments were analyzed: (1) to identify the DEGs responsible for each N treatment per genotype (N treatment effect) and (2) to identify the DEGs unique to the Pokkali per N condition (genotype effect). Analyses were performed for the following pair-wise comparisons: (1) Pokkali-low N (PKLN) vs. Bengal-low N (BGLN), (2) PKLN vs. Pokkali-full N (PKFN), (3) BGLN vs. Bengal-full N (BGFN), (4) Pokkali 1-h after transfer to full N (PK1H) vs. Bengal 1-h after transfer to full N (BG1H), (5) PK1H vs. PKLN, and (6) BG1H vs. BGLN. To identify the DEGs specific to various N treatments on each genotype, PKLN vs. PKFN and BGLN vs. BGFN were used for low nitrogen responses, and PK1H vs. PKLN and BG1H vs. BGLN for early N responses. To obtain the DEGs between Pokkali and Bengal under various LN and 1H treatments, PKLN vs. BGLN and PK1H vs. BG1H were used, respectively.

For gene ontology and plant pathway analyses, sets of DEGs were clustered in the following manner. For low N responses, DEGs from PKLN vs. PKFN, BGLN vs. BGFN, and PKLN vs. BGLN were combined. For early responses or 1-h N recovery periods, DEGs from PK1H vs. PKLN, BG1H vs. BGLN, and PK1H vs. BG1H were pooled. Gene ontology enrichment of the DEGs was performed to determine the biological significance with respect to the biological processes, molecular functions, and cellular localization of their proteins using a singular enrichment analysis (SEA) with agriGO v2 [78]. In addition, plant pathway analyses were performed using Plant Reactome (https://plantreactome.gramene.org) to determine the involvement of DEGs in plant pathways or processes [79].

### 4.9. Transcription Factors among DEGs

Transcription factors were identified and classified using the PlantTFDB v5.0, a plant transcription factor database [80]. The transcription factors among DEGs from the low N responses in Pokkali (PKLN vs. PKFN) and Bengal (BGLN vs. BGFN), and early N responses in Pokkali (PK1H vs. PKLN) and Bengal (BG1H vs. BGLN) were identified.

### 4.10. DEGs Co-Localized in Root Development-Related, ChloratE-Resistance, and NUE QTLs

DEGs identified as specific to N responses in Pokkali were co-localized with earlier reported QTLs for chlorate resistance, root development, and NUE. For potassium chlorate resistance (kclo_3_rs) and root development traits (root length: rtlg, root thickness: rtth, root volume: rtvol, root depth: rtdp and root number: rtnb), the Gramene QTL database [55] was used. For NUE-related traits, percent nitrogen content (*qNCP-3-1*), nitrogen use efficiency (*qNUE-3*) [58], and nitrogen response (*qNR4* and *qNR6*) [81] were considered. In some cases, DEG co-localization with QTLs was not possible due to a lack of information on physical chromosomal positions (Table S7). Co-localized DEGs for each QTL were illustrated using MapChart 2.3 [82].

### 4.11. Validation of Expression of NUE-Related Genes: qRT-PCR

The qRT-PCR was done following the protocol used in our laboratory [83] to validate the gene expression patterns obtained from the RNA-seq analysis. A total of 1 μg DNase-treated RNA from pooled biological replicates was used to synthesize cDNA with an iScript cDNA Synthesis Kit (Bio-Rad Laboratories, Inc., Hercules, CA, USA). The elongation factor 1 alpha (EF1α, *LOC_Os03g08010*) was used as an internal control for expression normalization in different cDNA samples. The expression analysis was done for the following genes: WRKY55 (*LOC_Os03g20550*), transcription factor BIM2 (*LOC_Os09g29930*)*,* ammonium transporter (*LOC_Os02g40730*), POT family protein/TGF-beta receptor (*LOC_Os05g34000*)*, NIGT1* (*LOC_Os02g22020*), polygalacturonase inhibitor precursor (*PGIP1* (*LOC_Os05g01380*))*,* ZIP3 (*LOC_Os04g52310*)*, NRT2.1* (*LOC_Os02g02170*), high-affinity urea transporter *DUR3* (*LOC_Os10g42960*)*,* Pectinesterase inhibitor (*LOC_Os12g18560*)*,* and MYB family transcription factor (*LOC_Os05g50340*). The reactions were set up in 96-well plates on an Applied Biosystems QuantStudio 3 Real-Time PCR system using iTaq™ Universal SYBR Green Supermix (Bio-Rad Laboratories, Inc) in a total reaction volume of 10 μL. The reactions were performed in three technical replicates using cDNA synthesized from pooled biological replicates and gene-specific primers designed using the PrimerQuest Tool (Integrated DNA Technologies, Inc., Coralville, IA, USA (Table S8)). The qRT-PCR expression levels of genes were determined using the 2^–ΔΔCT^ method as described earlier [84]. The Pearson correlation coefficients were calculated on the expression data of genes obtained from both qRT-PCR and RNA-seq analysis using the R (v 4.0.1) package *ggpubr* with *ggscatter* function.

## Figures and Tables

**Table 1:** Root architecture measurements of Pokkali and Bengal in low and full N.

Genotype	Treatment	Average Lateral Root Length (cm)	Average Lateral Root Diameter (cm)	Total Lateral Roots (range)	Total Surface Area (cm^2^)	Total Volume (cm^3^)
Bengal	Full N	8.20 ± 0.29 ^a,b^	0.029 ± 0.003 ^a^	5–8	4.19 ± 0.23 ^a^	0.036 ± 0.003 ^a^
	Low N	9.09 ± 0.56 ^a^	0.032 ± 0.003 ^a^	7–9	7.40 ± 0.13 ^b^	0.089 ± 0.003 ^b^
Pokkali	Full N	7.61 ± 0.40 ^b^	0.039 ± 0.001 ^b^	6–10	5.30 ± 0.33 ^c^	0.054 ± 0.006 ^c^
	Low N	11.60 ± 0.46 ^c^	0.036 ± 0.001 ^a,b^	8–13	9.67 ± 0.45 ^d^	0.107 ± 0.006 ^d^

Means in each column followed by the same letter are not significantly different (*p* < 0.05).

**Table 2:** Distribution of differentially expressed genes (%) in Plant Reactome pathway analysis.

Major Pathway	Pathway Name	Low N (%) ^a^	1-h after Full N (%) ^b^
Cellular process	DNA replication: activation of the pre-replicative complex	-	1.4
	Protein metabolism: translation	3.0	7.1
Growth and developmental process	Reproductive structure development	5.0	5.7
	Vegetative structure development	3.0	-
Metabolism and regulation	Amine and polyamine biosynthesis	1.0	-
	Amino acid metabolism	13.0	18.6
	Carbohydrate metabolism	8.0	5.7
	Cofactor biosynthesis	9.0	18.6
	Cytokinin 7-N-glucoside biosynthesis	2.0	1.4
	Cytokinin 9-N-glucoside biosynthesis	2.0	1.4
	Detoxification	2.0	-
	Fatty acid and lipid metabolism	2.0	-
	Generation of precursor metabolites and energy	2.0	-
	Hormone signaling, transport, and metabolism	25.0	21.4
	Inorganic nutrients metabolism	7.0	7.1
	Photorespiration	2.0	-
	Secondary metabolism	9.0	7.1
Response to stimuli: abiotic and stimuli and stresses	Response to cold temperature	2.0	2.9
	Response to heavy metals	1.0	1.4
	Response to phosphate deficiency	1.0	-
Response to stimuli: biotic and stimuli and stresses	Recognition of fungal and bacterial pathogens and immunity response	1.0	-

Distribution shows percentage of differentially expressed genes (DEGs; |log_2_ fold change| ≥ 2.0 and *padj* < 0.01). ^a^ All low N DEGs (PKLN vs. BGLN, PKLN vs. PKFN, BGLN vs. BGFN); ^b^ all 1-h treatment DEGs (PK1H vs. BG1H, PK1H vs. PKLN, BG1H vs. BGLN).

**Table 3:** Nitrogen uptake and metabolism-related DEGs of Pokkali and Bengal in response to low N and early (1-h) recovery conditions.

Function	MSU ID	Description	Low N	Early N Recovery	Low N	Early N Recovery
			Pokkali		Bengal	
Nitrate transporters	LOC_Os10g40600	Nitrate transporter 1.1B	-	U	-	U
	LOC_Os02g02170	High-affinity nitrate transporter 2.1	U	U	-	U
	LOC_Os02g02190	High-affinity nitrate transporter 2.2	U	U	U	U
	LOC_Os01g50820	High-affinity nitrate transporter 2.3	U	-	U	-
Ammonium transporter	LOC_Os02g40730	Ammonium transporter 1 member 2	D	U	-	U
Nitrate reductase	LOC_Os08g36480	Nitrate reductase 1	-	-	-	U
	LOC_Os08g36500	Nitrate reductase 2	-	-	-	U
	LOC_Os02g53130	NADH/NADPH-dependent NO3-reductase 2	D	U	D	U
Glutamine synthetase	LOC_Os03g12290	Glutamine synthetase 1;2	D	U	D	U
GOGAT	LOC_Os01g48960	NADH-GOGAT	-	U	-	U

U: upregulated; D: downregulated; ‘-‘: not differentially expressed.

**Table 4:** Numbers of transcription factors (TFs) differentially expressed in Pokkali and Bengal under low N and early (1-h) recovery conditions.

TF Family	Description	Low N	Early N Recovery	Low N	Early N Recovery
		Pokkali		Bengal	
AP2	AP2 family protein	1	0	0	0
B3	B3 family protein	0	0	3	1
bHLH	basic/helix-loop-helix family proteins	10	5	6	7
bZIP	bZIP family protein	3	6	0	6
C2H2	C2H2 zinc finger domain	9	6	5	3
C3H	Cys3His -containing zinc finger domain	0	1	0	0
CO-like	CO (CONSTANS) family protein	1	1	0	1
Dof	Dof (DNA binding with one finger) family	2	0	0	0
EIL	Ethylene-insensitive3 (EIN3) and EIN3-like (EIL) family proteins	0	2	0	1
ERF	ERF family protein	9	18	8	15
FAR1	FAR1 family protein	1	0	1	1
G2-like	G2-like family protein	5	3	1	0
GATA	GATA family protein	2	0	2	1
GRAS	GRAS family protein	1	0	1	1
GRF	GROWTH-REGULATING FACTOR family protein	0	1	0	1
HD-ZIP	HD-ZIP family protein	0	3	3	1
HSF	Heat stress transcription factors	1	1	0	1
LBD	LBD family protein	2	5	3	4
MIKC-MADS	MIKC-MADS family protein	1	0	1	0
M-type MADS	M-type MADS family protein	0	0	0	1
MYB	MYB family protein	8	1	4	4
MYB-related	MYB-related family protein	3	2	2	2
NAC	NAM, ATAF, and CUC (NAC) transcription factors	8	3	7	3
NF-YA	NF-YA family protein	2	0	2	0
NF-YB	NF-YB family protein	0	1	0	1
Nin-like	Nin (for nodule inception)-like family protein	1	1	1	0
RAV	RAV family protein	0	1	0	1
SBP	SQUAMOSA promoter binding proteins (SBPs)	1	0	1	0
WOX	WOX family protein	0	1	0	0
WRKY	WRKY family protein	17	2	9	9
	Total	88	64	60	65

## Supplementary Materials

Supplementary Materials can be found at https://www.mdpi.com/1422-0067/21/16/5759/s1.

## Abbreviations

N	Nitrogen
NUE	Nitrogen use efficiency
DEG	Differentially expressed genes
QTL	Quantitative trait loci
TF	Transcription factor
PK	Pokkali
BG	Bengal
PKFN	Pokkali-Full nitrogen
PKLN	Pokkali-low nitrogen
PK1H	Pokkali-1h after transfer from low N to full N
BGFN	Bengal-full nitrogen
BGLN	Bengal-low nitrogen
BG1H	Bengal-1h after transfer from low N to full N
GO	Gene ontology
SEA	Singular enrichment analysis
CEP	C-terminally encoded peptides
CLE	CLV3/ESR-related
rtnb	Root number
rtlg	Root length
rtth	Root thickness
rtvol	Root volume
rtdp	Root depth
kclo3rs	Potassium chlorate resistance
AS	Alternate splicing
IR	Intron retention
A3SS	Alternative 3′ splice site
ES	Exon skipping
A5SS	Alternative 5′ splice site
MXE	Mutually exclusive exon
GOGAT	Glutamine oxoglutarate aminotransferase
qRT-PCR	Quantitative reverse transcription PCR
NIGT1	Nitrate-Inducible, GARP-type Transcriptional Repressor 1
GID1	GA insensitive dwarf1
NGR5	Nitrogen-mediated tiller growth response 5
PRC2	Polycomb repressive complex 2
