# Supplementary material for: Comparative Transcriptomics of Rice Genotypes with Contrasting Responses to Nitrogen Stress Reveals Genes Influencing Nitrogen Uptake through the Regulation of Root Architecture
Source: Int J Mol Sci. 2020 Aug 11;21(16):5759. doi: 10.3390/ijms21165759 (PMC7460981; doi:10.3390/ijms21165759)
Supplement: Supplementary file 1 [file ijms-21-05759-s001.zip › ijms-876564 Suppl Figs .pdf]

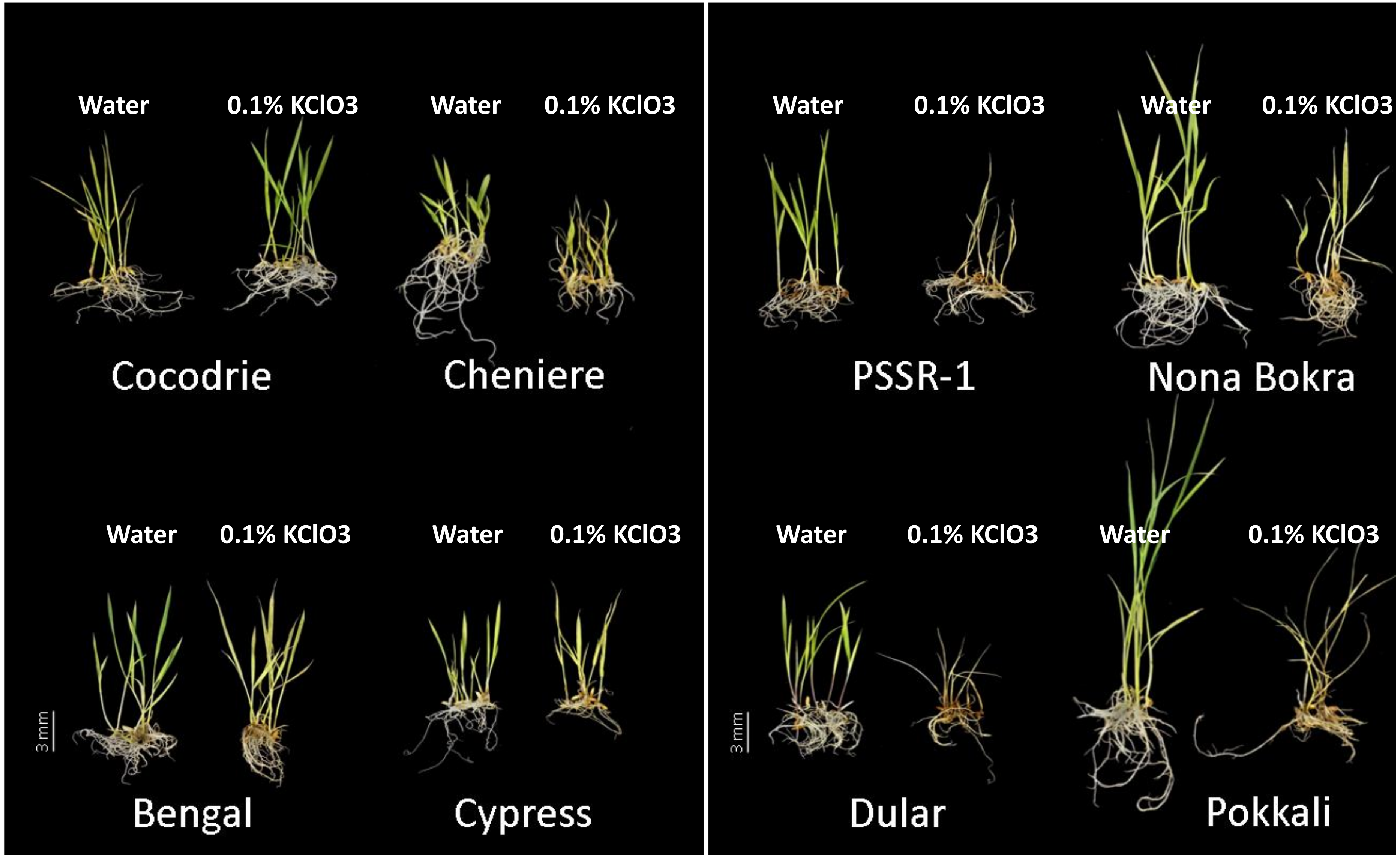

Figure S1. Effect of chlorate uptake on plant height in eight rice genotypes (Cocodrie, Bengal, Cheniere, Cypress, PSRR-1, Dular, Nona Bokra, and Pokkali after 5-day treatment with 0.1% $KCl0_3$. Scale bar is 3 mm.

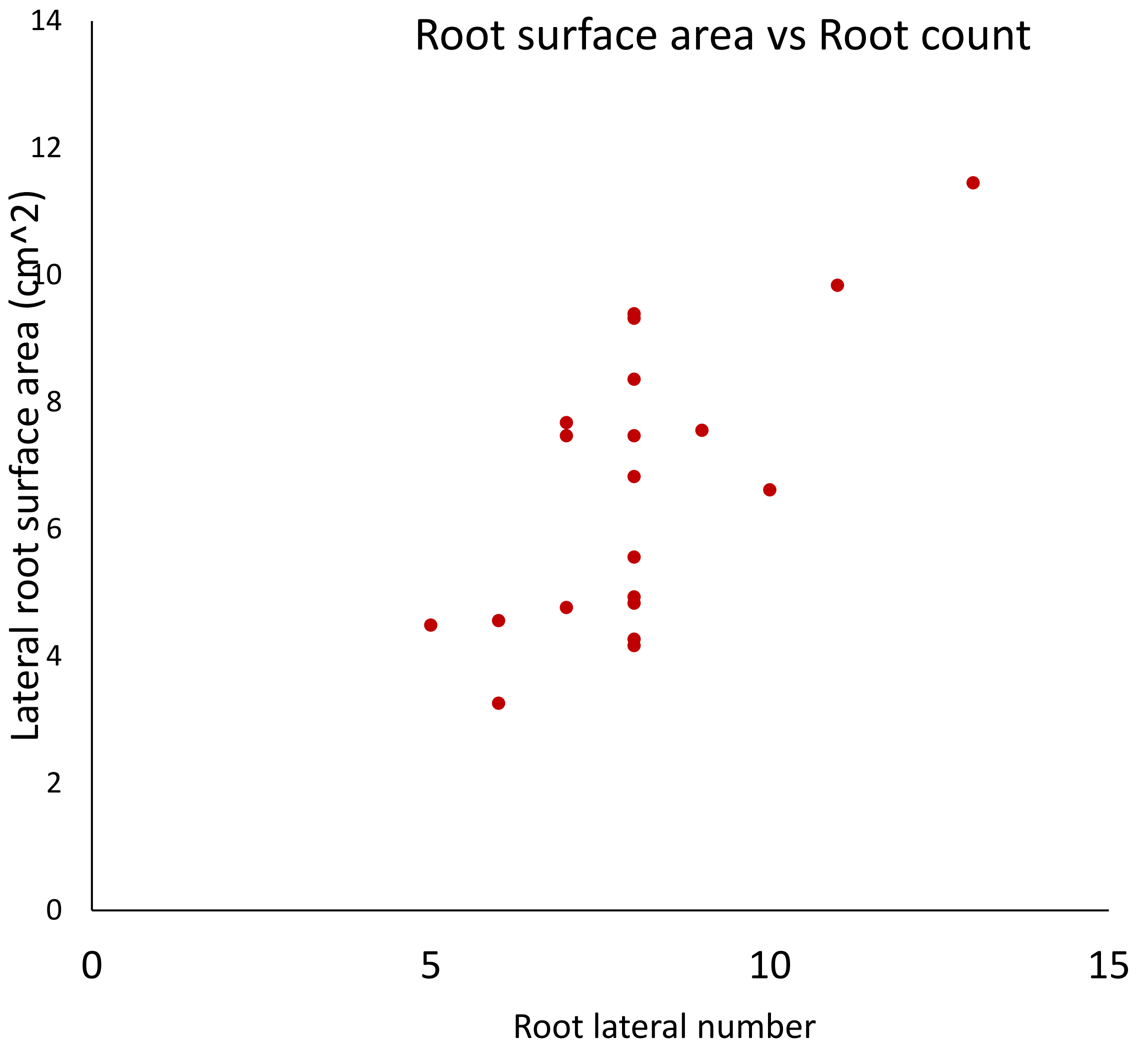

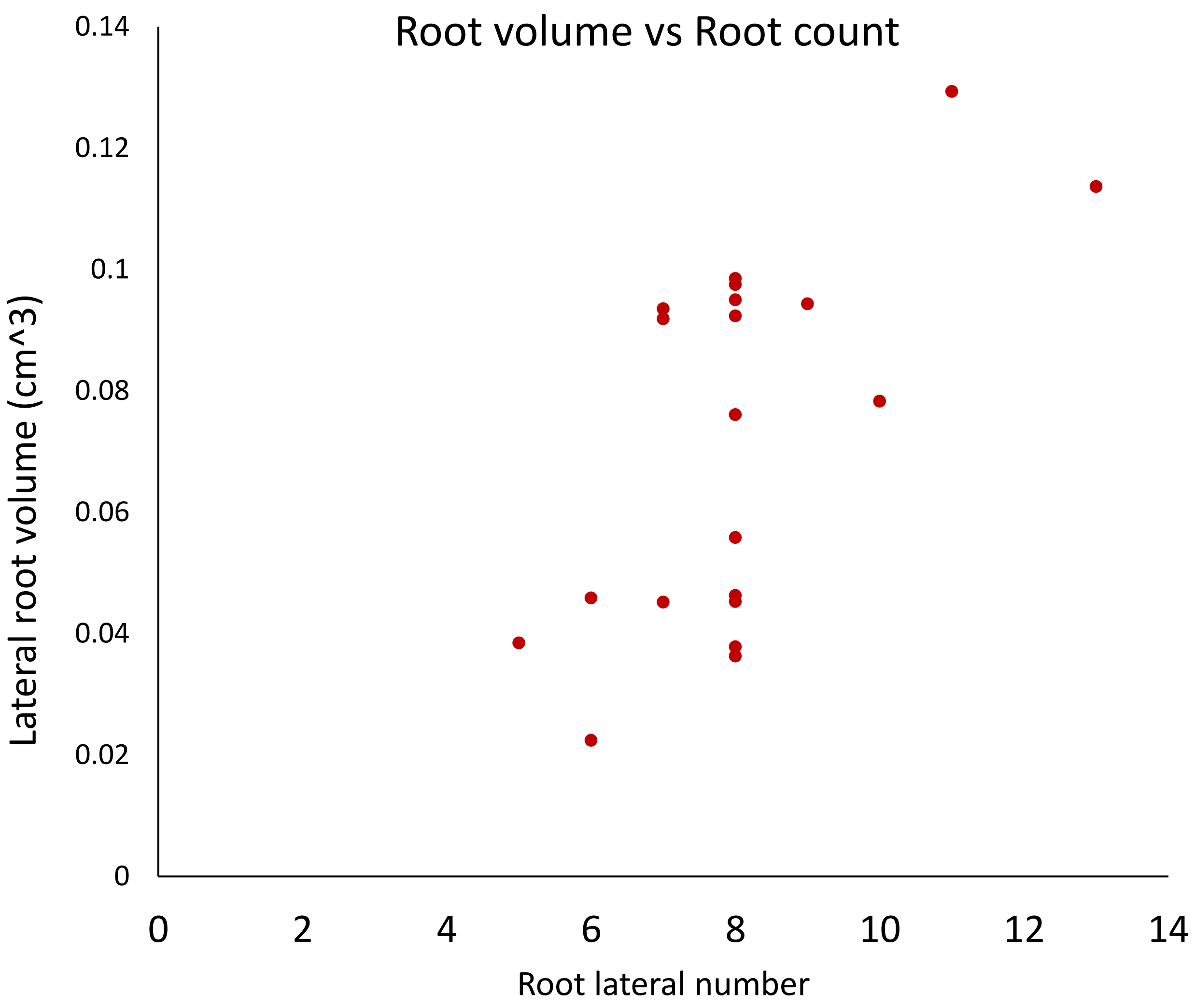

Pearson's correlation: 0.6851
p-value: 0.0009

Pearson's correlation: 0.6376
p-value: 0.0025

Figure S2. Pearson correlations and p-value of root surface area and volume versus root count.

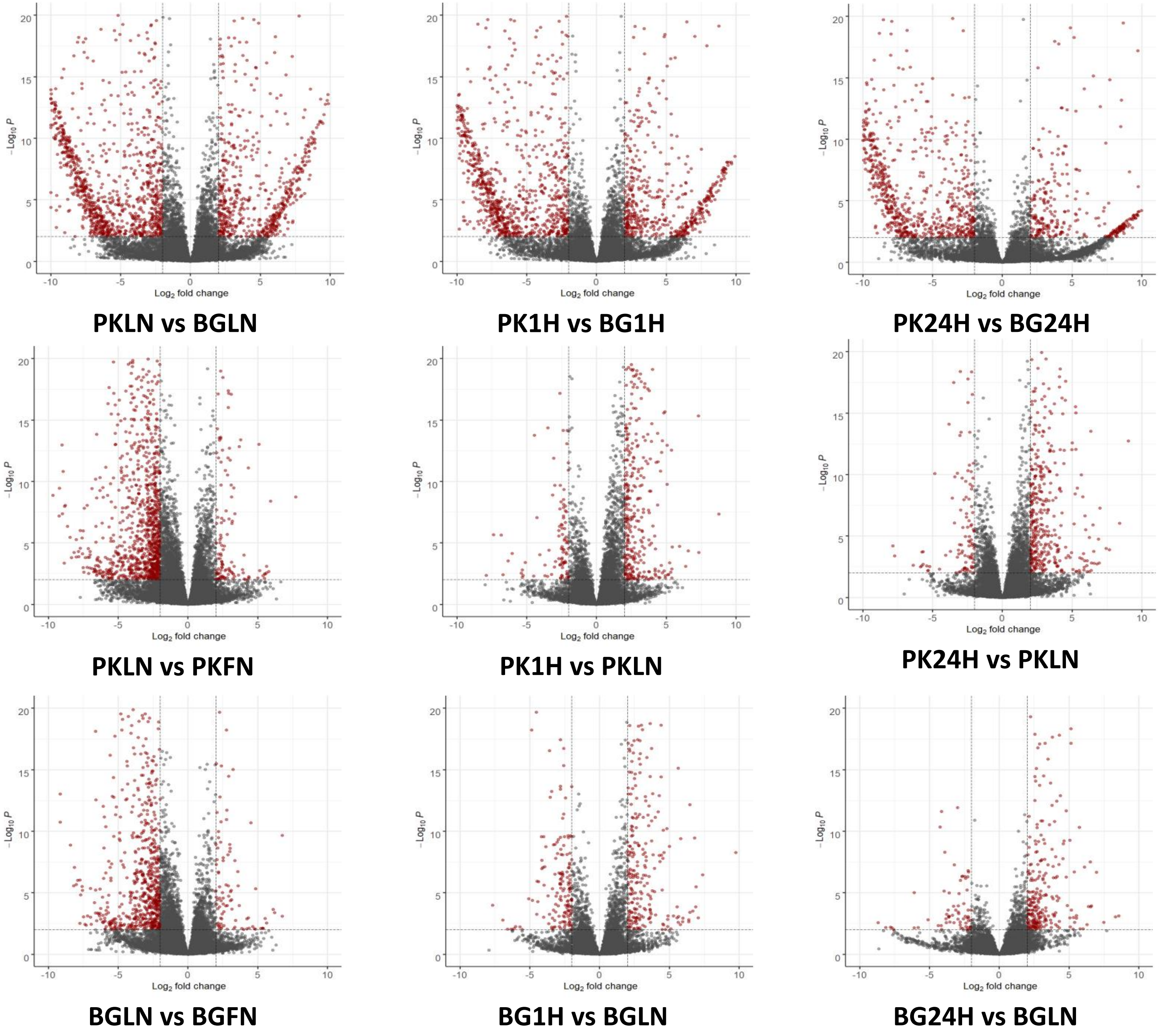

Figure S3. Volcano plot of multiple pairwise comparison between Pokkali and Bengal at low nitrogen, 1h after full nitrogen, and 24h after full nitrogen. Differentially expressed genes are shown as red dots. Gray dots represent genes that did not pass the cut-off point of $|\log_2 \text{fold change}| \geq 2$ and $padj < 0.01$. Plot was visualized using Bioconductor (release 3.10) package “EnhancedVolcano”.

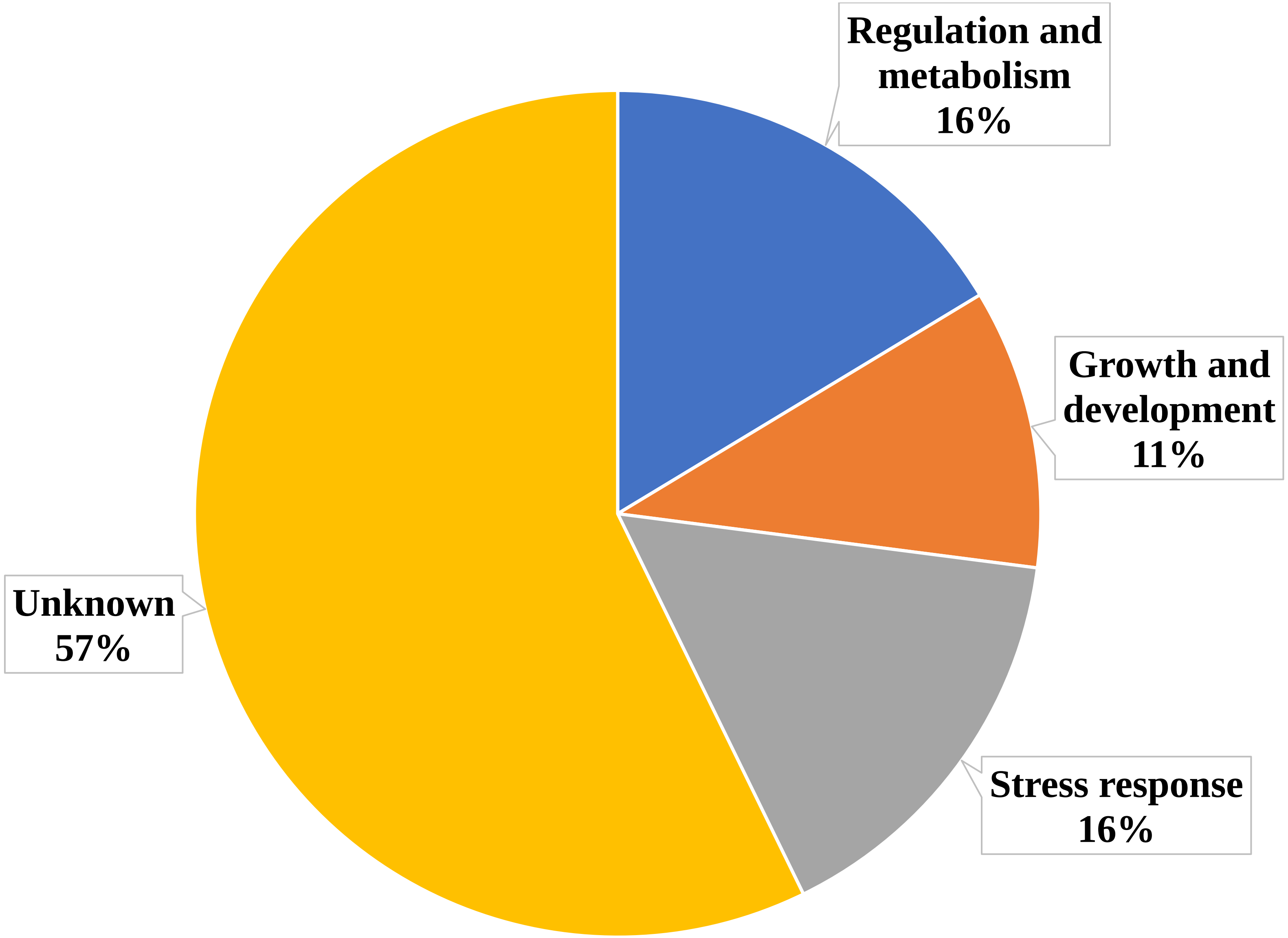

Figure S4. Gene ontology functional classification of root Pokkali-specific DEGs under various N treatments.

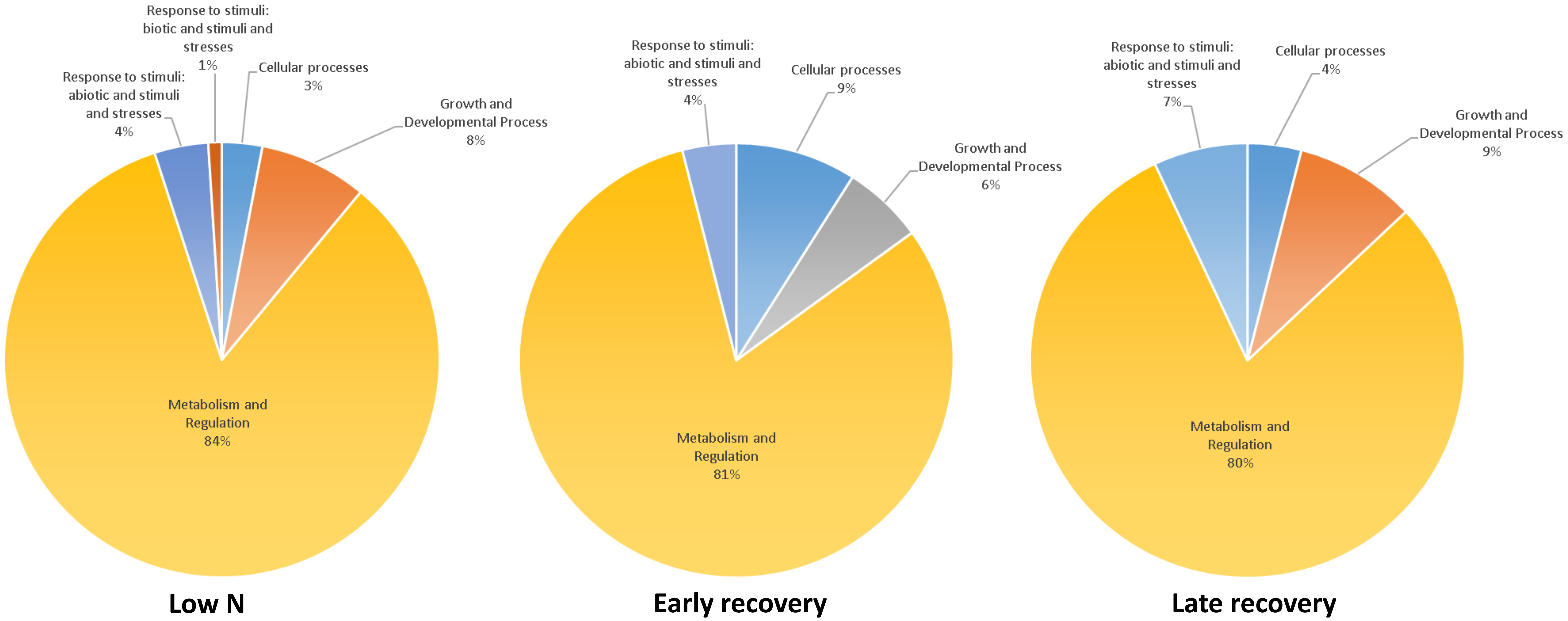

Figure S5. Distribution of DEGs according to the plant reactome pathway analysis. Differentially expressed genes obtained from combined data of low N (PKLN vs PKFN, PKLN vs BGLN and BGLN vs BGFN), 1-H response/early N response (PK1H vs PKLN, PK1H vs BG1H and BG1H vs BGLN), and 24-H response/late N response (PK24H vs PKLN, PK24H vs BG24H, and BG24H vs BGLN) were considered for this analysis.

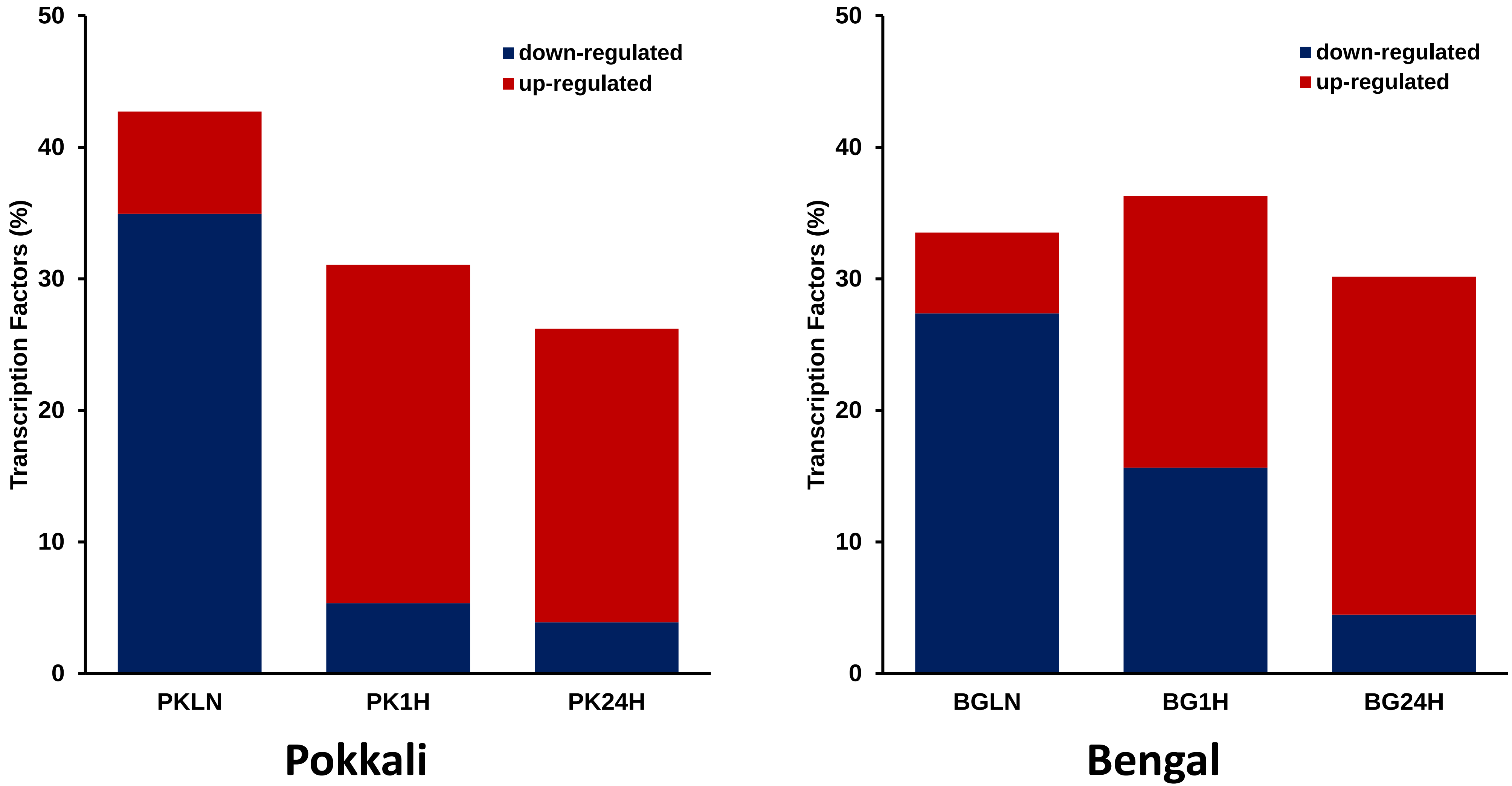

Figure S6. Regulation pattern of transcription factors in Pokkali (n=206) and Bengal (n=179) in different N conditions. Differentially expressed TF genes obtained from combined data of low N (PKLN vs PKFN, PKLN vs BGLN and BGLN vs BGFN), 1-H response/early N response (PK1H vs PKLN, PK1H vs BG1H and BG1H vs BGLN), and 24-H response/late N response (PK24H vs PKLN, PK24H vs BG24H, and BG24H vs BGLN). Red bars indicate up-regulated TFs while blue bars are down-regulated TFs.

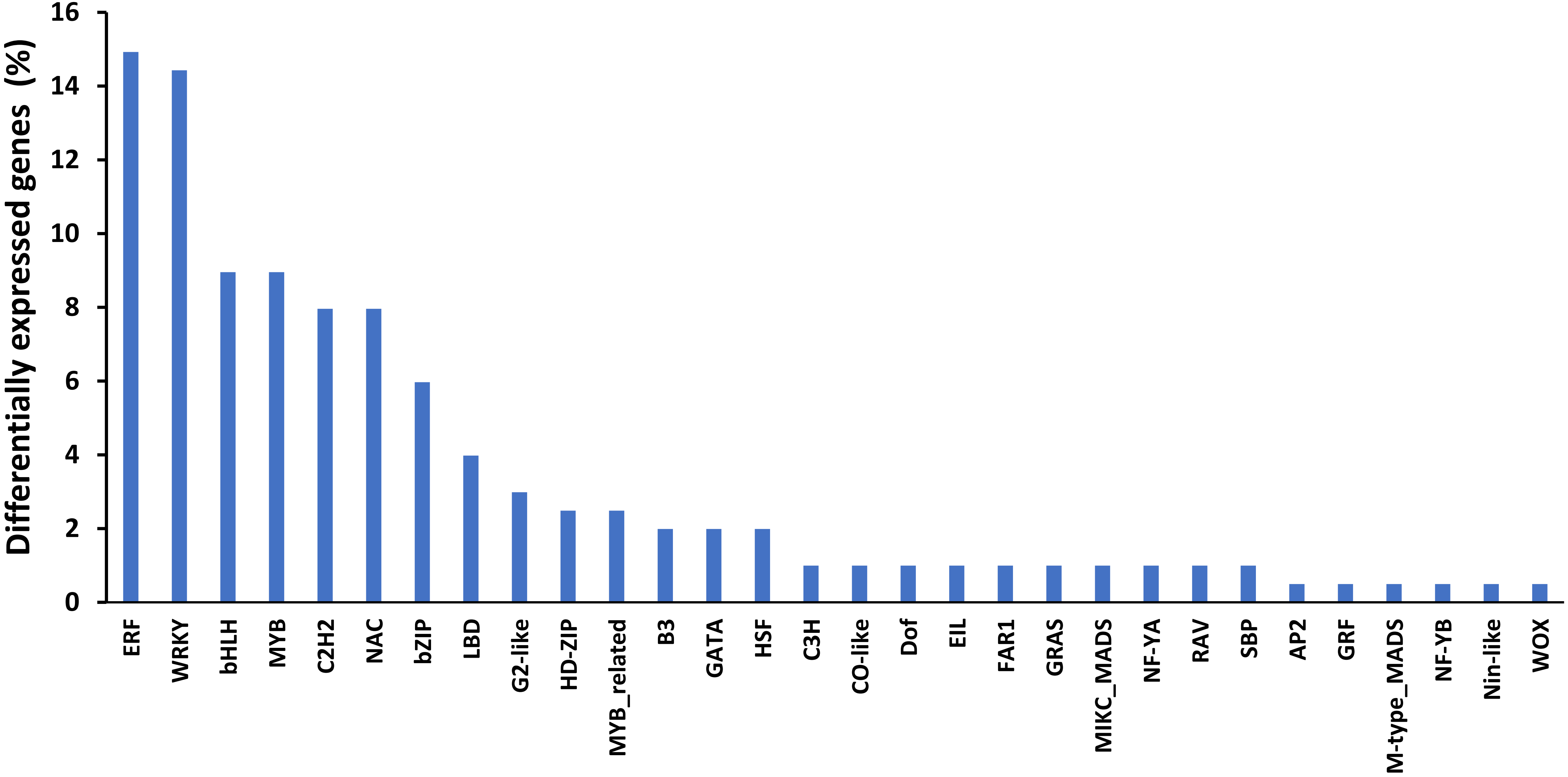

Figure S7. Percent of differentially expressed genes (DEGs) identified per transcription factor family (combined DEGs from all N conditions).

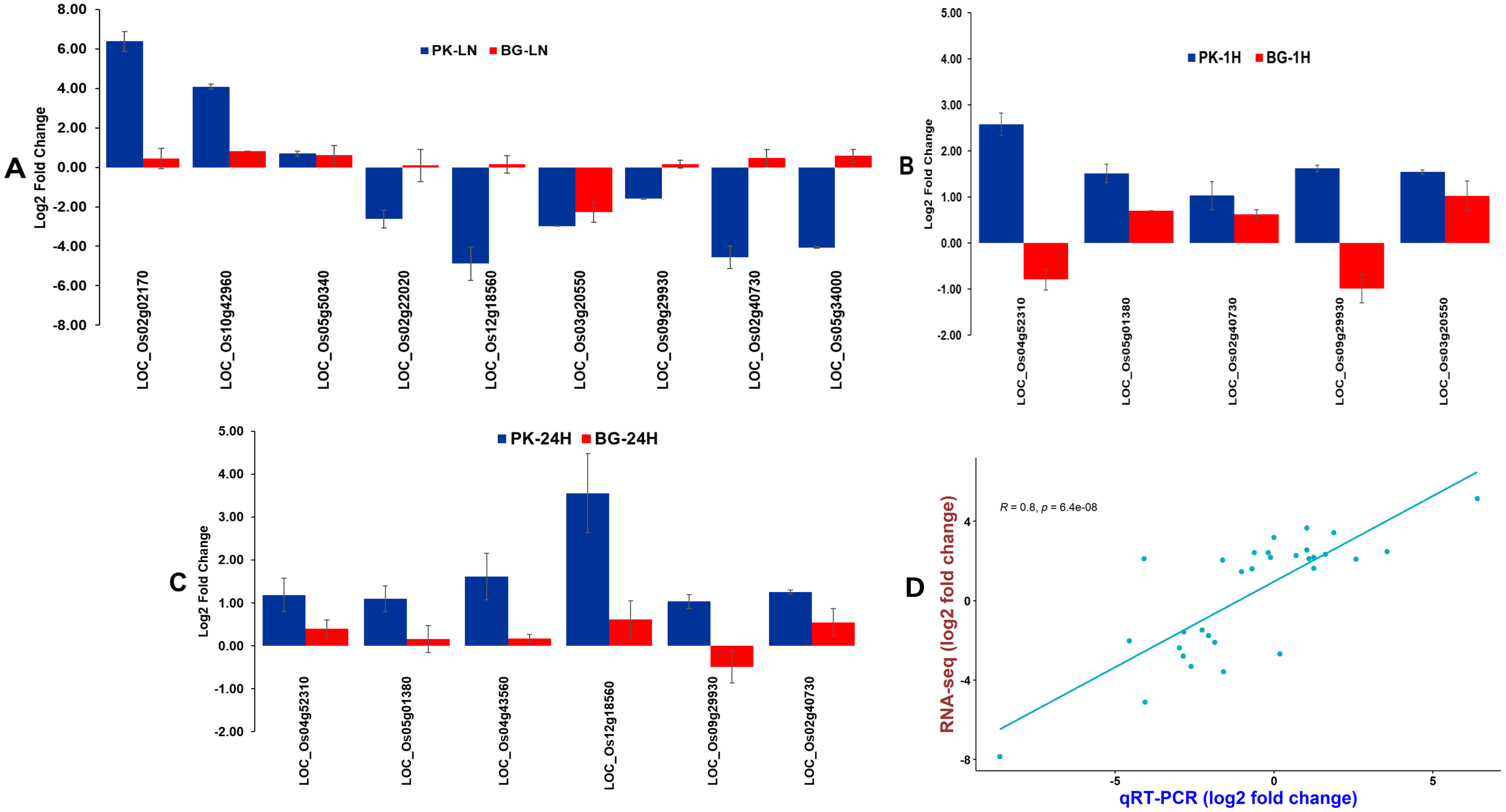

Figure S8. Validation of RNA-seq data *via* qRT-PCR. Bar plot showing relative transcript abundance of selected genes in Bengal and Pokkali under different N treatments are shown. *EF1α* was used as the reference gene. Pokkali/Bengal in full nitrogen was used as reference sample for low nitrogen stress (PK/BG-LN) (A). Pokkali/Bengal in low nitrogen (PK/BG-LN) was used as reference sample for 1 h and 24 h recovery treatments, respectively (B and C). Pearson correlation analysis between RNA-seq and qRT-PCR results is shown (D). Each dot (blue green) represents log_2 fold change for the selected genes in each corresponding sample (12 genes and 8 samples). Solid blue line is the line of best fit.
