# Supplementary material for: Comparative Transcriptomics of Rice Genotypes with Contrasting Responses to Nitrogen Stress Reveals Genes Influencing Nitrogen Uptake through the Regulation of Root Architecture
Source: Int J Mol Sci. 2020 Aug 11;21(16):5759. doi: 10.3390/ijms21165759 (PMC7460981; doi:10.3390/ijms21165759)
Supplement: Supplementary file 1 [file ijms-21-05759-s001.zip › Supplementary Tables_Aug 10 PS Edit(1).pdf]

Table S1. Pearson correlation coefficients among the root parameter. *p*-values are given in parenthesis.

Traits	Root length	Root surface area	Root volume	Root diameter
Root length	1.000			
Root surface area	0.7471 (0.0000)	1.000		
Root volume	0.5053 (0.0015)	0.9458 (0.000)	1.000	
Root diameter	-0.0401 (0.8163)	0.6111(0.0001)	0.7984(0.000)	1.000

Table S2. Summary of RNA-Seq assembly results in Bengal and Pokkali in each treatment.

Samples§	Assembled Reads	Total Reads	Assembled Reads (%)	Total no. of Transcripts	Novel Transcripts (loci)
BGFN	109,966,765	131,910,063	83	67980	2320 (1857)
BGLN	85,872,294	133,491,930	64	72865	3262 (2587)
BG1H	98,666,440	156,256,445	63	67874	2376 (2300)
PKFN	108,556,018	126,936,285	86	67366	2190 (1763)
PKLN	101,225,496	130,590,725	78	71661	2621 (2056)
PK1H	116,498,065	136,862,915	85	69311	2303 (1874)

§Data in first three rows are from three treatments of Bengal and last three rows are from Pokkali. Each row refers to data from different treatments pooled over all replicates. The treatment groups are BGFN (Bengal-Full N), BGLN (Bengal-low N), BG1H (Bengal-1h-after transfer from low N to full N), PKFN (Pokkali-Full N), PKLN (Pokkali-low N), PK1H (Pokkali-1h-after transfer from low N to full N).

Table S4. Long distance signaling DEGs of Pokkali and Bengal in response to various N conditions.

Signaling protein[$]	MSU ID	Description	Low N	Early N recovery	Low N	Early N recovery
			Pokkali		Bengal	
CEPs	LOC_Os05g11620	C-Terminally Encoded Peptide 11	D	U	-	-
	LOC_Os05g11580	C-Terminally Encoded Peptide 12	-	U	-	U
	LOC_Os06g43080	C-Terminally Encoded Peptide 15	-	-	D	-
CLEs	LOC_Os01g23705	CLV3/ESR-related 102	U	-	-	-
	LOC_Os01g32560	CLV3/ESR-related 103	-	D	-	-
	LOC_Os03g48570	CLV3/ESR-related 305	-	-	-	D

[$]CEP, C-terminally encoded peptides; CLE, CLV3/ESR-related

U: up-regulated; D: down-regulated; '-': not differentially expressed.

Table S5. Total number of co-localized DEGs with QTL governing NUE, root morphology, and chlorate-resistance.

Trait	All Low N-DEGs	Pokkali specific-Low N DEGs
NUE	79	1
Chlorate resistance	483	20
Root thickness	1256	39
Root length	1080	24
Root number	1774	55
Root volume	167	2
Root depth	96	2

Table S6. Alternative splicing events present in Pokkali-specific differentially expressed genes compared with Bengal in different treatments.

Locus	BGFN[a]	BGLN[a]	BG1H[a]	PKFN[a]	PKLN[a]	PK1H[a]
LOC_Os03g16070	-	-	A5SS|A3SS	A3SS	A3SS	-
LOC_Os03g47754	A3SS	-	-	A3SS	-	-
LOC_Os03g62060	IR	A3SS	-	A3SS	-	-
LOC_Os04g30420	-	-	-	A3SS	-	-
LOC_Os05g15530	-	-	IR	-	-	-
LOC_Os06g40415	A3SS	A3SS|A5SS + A3SS	A3SS	-	-	-
LOC_Os06g43780	IR	-	-	A3SS	-	-
LOC_Os06g48160	-	-	A5SS	-	-	-
LOC_Os07g32570	-	IR	-	-	-	-
LOC_Os07g36390	-	IR	-	-	-	-
LOC_Os07g43440	-	ES	-	-	-	ES
LOC_Os09g08130	-	A3SS	-	-	-	-
LOC_Os09g19734	IR1 + IR2|IR|A3SS	IR1 + IR2|IR	IR1 + IR2|IR	IR1 + IR2|IR	IR1 + IR2|IR	IR1 + IR2|IR
LOC_Os09g29930	-	-	-	A5SS or A3SS	A3SS	ES
LOC_Os11g02670	-	A3SS	-	-	-	-
LOC_Os11g08100	-	A5SS	-	-	-	A5SS
LOC_Os11g40690	IR	IR	IR	IR	A3SS|IR	IR

[a] Merged transcript assembly of replicates per genotype within a treatment was used in AS analysis.
Treatments were: BGFN, Bengal-Full N; BGLN, Bengal-low N; BG1H, Bengal-1h after transfer from low N to full N; PKFN, Pokkali-Full N; PKLN, Pokkali-low N; PK1H, Pokkali-1h after transfer from low N to full N.

Table S7. Published QTLs governing NUE. For chlorate resistance (potassium chlorate resistance: kclo3rs) and root development traits (root length: rtlg, root thickness: rtth, root volume: rtvol, root depth: rtdp, and root number: rtnb), Gramene QTL database (Ni et al. 2009) [55] was used.

Trait	QTL	Chr	Marker interval	Position of first marker	Position of second marker	LOD	Var %	Reference
Nitrogen content (%) (NCP)	qNCP-3-1	3	RG191–RZ678	5,729,669-5,730,162	7,347,629-7,347,996	6.12	29.1	Senthilvel et al. (2008)
Nitrogen content (%) (NCP)	qNCP-3-2	3	Pgi1– CDO87	NA	NA	4.67	23.6	Senthilvel et al. (2008)
Nitrogen use efficiency (NUE)	qNUE-3	3	RZ574 – RZ284	10,599,177-10,599,382	15,469,002-15,469,518	5.46	26.4	Senthilvel et al. (2008)
Nitrogen response (NR)	qNR6	6	RZ398-C764	3,459,492-3,459,750	2,770,460-2,771,568	5.48	16.6	Wei et al. (2012)
Nitrogen response (NR)	qNR4	4	G235-G102	31,471,245-31,471,486	30,334,770-30,334,896	3.65	11.01	Wei et al. (2012)
Physiological NUE (PNUE)	qPE3	3	RM232-C63	NA	8,409,404-8,410,886	4.14	12.8	Wei et al. (2012)
Physiological NUE (PNUE)	pnue9	9	S9093-S9073	NA	NA	4.62	15.3	Cho et al. (2007)

NA, Not available

Table S8. List of primers used for expression validation by qRTPCR.

Gene	Locus ID	Forward Primer (5'-3')	Reverse Primer (5'-3')
Invertase/pectin methylesterase inhibitor family protein	*LOC_Os12g18560*	GGAGAGAAACACCAATGGC	CGTGCTTGGAAGAAGGC
MYB family transcription factor (NIGT1)	*LOC_Os02g22020*	CAAGGACAGAGACATTCCG	ACGAATCTACACTAATCAAGCG
Polygalacturonase inhibitor precursor (PGIP1)	*LOC_Os05g01380*	TCCTACAAGAACAAGATGCG	GACGTCGTACCATTCGC
Metal cation transporter (ZIP3)	*LOC_Os04g52310*	CTAGTTGACCTTCTAGCAACAG	TCAAGCCTATCTGGGAAATAAG
MYB family transcription factor	*LOC_Os05g50340*	AGCAGAACAAGCTGTTCG	TCCTCCAGCATCTCGTAGTA
Urea active transporter (DUR3)	*LOC_Os10g42960*	TCCATCTATCCGTCTGTTAGAG	CGTTCACCACCGAGTAGTA
Transporter, major facilitator family (NRT2.1)	*LOC_Os02g02170*	CGATTCCATCTCCAAGAAGC	CCACCTCCGTCGAGAAC
Transcription factor BIM2	*LOC_Os09g29930*	CCAGTACATCAGCGAACAAC	AGAGTGAGAACCGCCATC
WRKY55	*LOC_*Os03g20550	CGATTCCTGAAGAGACAGTAG	CATACTCTTTGAGGGTGTAGAG
POT family protein (TGF-BR)	*LOC_Os05g34000*	TCAACCTCGCCTACTAC	TGCTAGCTTACAACACC
Ammonium transporter protein	*LOC_Os02g40730*	CGGGTTCATGCTCAAGTC	TTCGTTCGATCGATGATGC
Elongation factor 1 alpha (EF1α)	*LOC_Os03g08010*	TTGATCTGGTCAAGAGCCTCAAGC	TCTCTGGGTTTGAGGGTGACAACA
